# Site-specific ion occupation in the selectivity filter causes voltage-dependent gating in a viral K^+^ channel

**DOI:** 10.1038/s41598-018-28751-w

**Published:** 2018-07-10

**Authors:** O. Rauh, U. P. Hansen, D. D. Scheub, G. Thiel, I. Schroeder

**Affiliations:** 1 Plant Membrane Biophysics, Technische Universität Darmstadt, Darmstadt, Germany; 2 Department of Structural Biology, Christian-Albrechts-Universität zu Kiel, Kiel, Germany

## Abstract

Many potassium channels show voltage-dependent gating without a dedicated voltage sensor domain. This is not fully understood yet, but often explained by voltage-induced changes of ion occupation in the five distinct K^+^ binding sites in the selectivity filter. To better understand this mechanism of filter gating we measured the single-channel current and the rate constant of sub-millisecond channel closure of the viral K^+^ channel Kcv_NTS_ for a wide range of voltages and symmetric and asymmetric K^+^ concentrations in planar lipid membranes. A model-based analysis employed a global fit of all experimental data, i.e., using a common set of parameters for current and channel closure under all conditions. Three different established models of ion permeation and various relationships between ion occupation and gating were tested. Only one of the models described the data adequately. It revealed that the most extracellular binding site (S0) in the selectivity filter functions as the voltage sensor for the rate constant of channel closure. The ion occupation outside of S0 modulates its dependence on K^+^ concentration. The analysis uncovers an important role of changes in protein flexibility in mediating the effect from the sensor to the gate.

## Introduction

Transport through ion channels can be controlled by membrane potential, e.g. during the excitation of neurons and muscle cells^1,2^. In Kv channels, voltage sensitivity is conferred by the voltage-sensing domain (VSD), which is coupled to the conserved K^+^ channel pore domain. The VSD consists of a tetramer of four transmembrane helices per subunit, one carrying 4–5 positive charges^3^. Its function is quite well understood^4,5^.

However, also channels without a VSD can exhibit voltage sensitivity. This is for example important in K2P channels, which modulate in this way their slow inactivation^6^. In addition to these slow events, which occur in a time window of several tens of ms, also voltage-sensitive gating events in the millisecond^7–10^ and microsecond range^11–14^ have been described. These fast events can cause a negative slope in the apparent single-channel current-voltage relationship (IV curve)^11,12,14^. The mechanism of voltage dependence without VSD is still vague. C-type inactivation seems to be related to the ion occupation in the selectivity filter^6,15–17^, and also in Ca^2+^-gated MthK channels, the voltage-dependent gate has been located in the filter^18^. It has been proposed that the positive charges of the transported ions compensate the repulsive forces of the negative charges of the carbonyl groups lining the selectivity filter. Voltage-induced ion depletion of the binding sites consequently modifies the conformation of the filter^19–22^ and thus modulates the rate constants of gating. So far, evidence for the relationship between ion occupancy and conformation has been provided by crystallographic^23,24^, electron cryomicroscopy (cryo EM)^25^ or nuclear magnetic resonance (NMR) studies^26–28^ and computational modeling^6,29–32^.

To test the predictions from these approaches it is desirable to determine the postulated changes in ion distribution directly from the same single-channel recordings, which are also used for the quantitative description of the voltage-dependent gating. For several reasons, the viral K^+^ channels of the Kcv family are very appropriate candidates for such a study. First, they have a high unitary conductance, which supports high-resolution single-channel analysis^33,34^. Second, they exhibit a distinct voltage sensitivity, resulting in a very pronounced negative slope of the apparent single-channel IV curve^14,33,34^. It has been shown that the related reduction of the apparent current results from averaging over a normally hidden gating process with dwell times in the closed state between 50 and 150 µs (sub-millisecond gating). The overall voltage dependence of this gating process corresponds to the transfer of one electrical charge through the whole electric field^14^. Third, the Kcv family has about 80 members with different functional characteristics caused by just a few different residues in the sequence^35^. This provides guidelines for efficient mutational studies^34^. Fourth, a channel monomer consists of only 80 to 120 residues^35^. The combination of small size, high unitary conductance and distinct gates increases the possibility of assigning each distinct gate to a defined molecular mechanism.

Here, we employ a novel approach to determine ion distribution in the selectivity filter from single-channel recordings using a model-based IV curve analysis^36^. This is encouraged by previous studies, where simple Markov models for ion transport comprising loading, translocation and recycling steps have yielded important insights such as the binding order in cotransporters^37^ or the effect of internal pH on H^+^ pump stoichiometry^38^. In bacteriorhodopsin, several models of transport could be distinguished^39^. In the influenza A proton channel M2, we could verify the rapid exchange of protons between cytosol and the His37 proton binding site, the resetting of the tilted helix after each translocation cycle, transinhibition of ion uptake by cytosolic H^+^ concentration, and the origin of rectification^40^. In KcsA, a “mesoscopic” approach has been suggested combining Markov models and structural information^41^.

Now, atomistic models of ion transport through the selectivity filter in K^+^ channels have become available^31,42,43^. This opens the access to the evaluation of the voltage-dependent ion distribution in K^+^ channels from functional data. Here, we use single-channel data to test three different models for K^+^ transport through the selectivity filter of the viral channel Kcv_NTS_. For this K^+^ channel, we have found that only the 5-state ion-hopping model of Roux^42^ is consistent with the data. It can fit the voltage and K^+^ dependence of the IV curves and of the rate constant of channel closure in a global fit. The results identify the voltage sensor of this gating process and indicate that a change in protein flexibility rather than in conformation provides the link between sensor and gate.

## Results

### The gating processes in Kcv_NTS_

**Figure 1:**
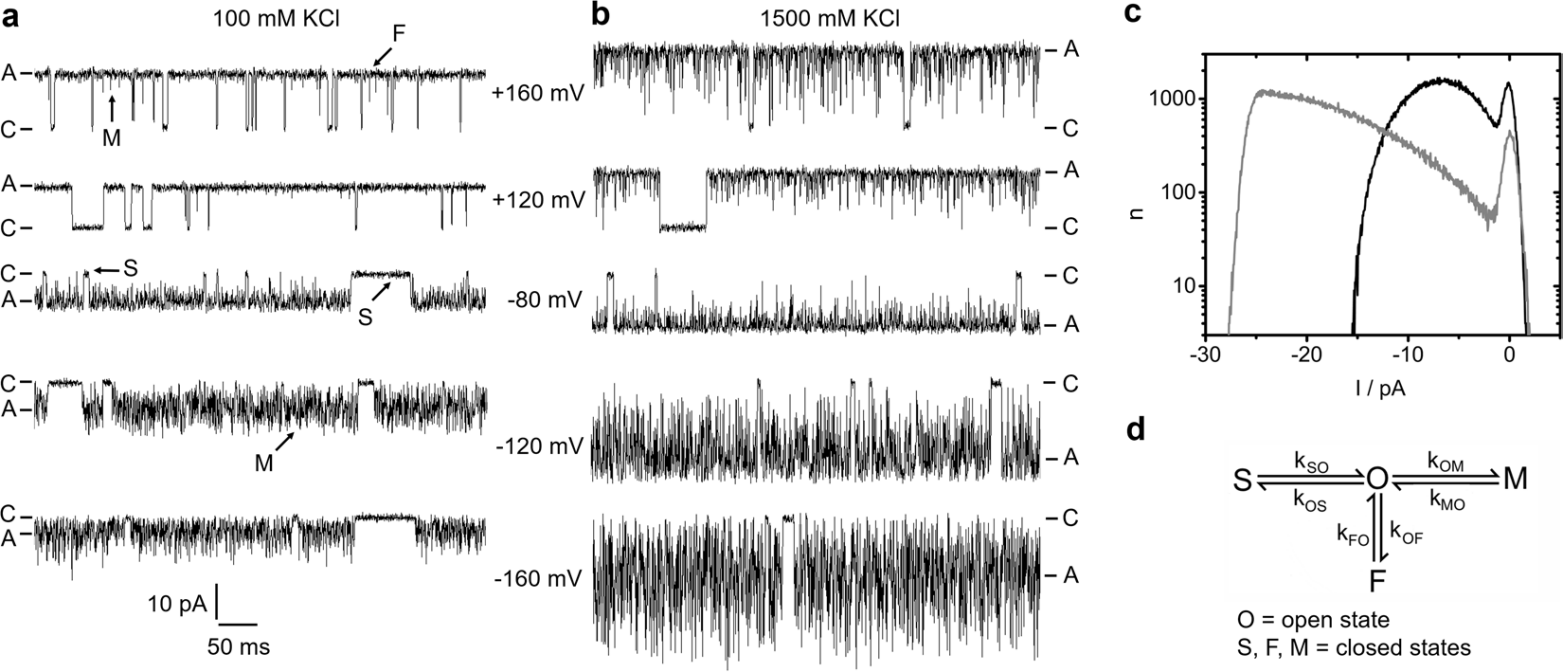
Gating of Kcv_NTS_. **(a**,**b)** Typical time series of current measured in symmetrical KCl solutions of **(a)** 100 mM and **(b)** 1500 mM. Closed “C” and apparent open level “A” are labelled. **(c)** Representative amplitude histograms in 100 (black) and 1500 mM KCl (grey), recorded at −120 mV. **(d)** Markov model of gating comprising one open state O, and three closed states (S = slow, M = medium, F = fast). The labels in (**a**) correspond to the states in (**d**).

Kcv_NTS_ shows several distinct gating processes; in the recordings in Fig. 1a,b, long closed events (“S”) as well as shorter events (“M”) are visible. The faster process, causing the large open channel noise at negative voltages is voltage-dependent and partially suppressed at higher K^+^ concentrations (compare the traces at −120 mV for 100 and 1500 mM) as indicated by the increased asymmetry of the noise.

In a previous paper^14^, it has been found that the Markov model in Fig. 1d is adequate for describing the dominant gating processes of this channel. Two slow gating processes (labelled by S) can be clearly revealed by dwell time analysis^34^, as they reach the full closed level. Since these slow gating events contribute equally to the amplitude histograms in Fig. 1c (the basis for the gating analysis below), they all are merged into the O-S transitions in Fig. 1d, with O being the open state. Unlike the slow gating, the faster processes are not resolved by full transitions to the closed state. The O-M gating with dwell times in the closed state M between 50 and 150 µs only causes small peaks in Fig. 1a (labelled by “M”). The recordings obtained at different membrane voltages show that O-M gating is strongly voltage-dependent. This is remarkable since Kcv channels do not have a VSD. The time series obtained at different K^+^ concentrations (Fig. 1a,b) reveal that O-M gating is also strongly sensitive to the K^+^ concentration in the solutions. The third gating process (O-F) is even faster with dwell times in the closed state F between approximately 1 to 10 µs. Its existence becomes obvious only from the broadening of the noise of the apparent open state (label “F” in Fig. 1a).

### The analysis of gating by extended beta distributions

Since O-M and O-F gating cannot be evaluated directly by dwell-time analysis or similar methods, we utilize extended beta distributions^44^. Fast gating events with rate constants even much higher than the cut-off frequency of the low-pass filter of the recording system cause increased open channel noise (Fig. 1a,b) and thus characteristic distortions of the current amplitude histograms (Fig. 1c). Thus, the fast rate constants can be determined by a fit algorithm adjusting them until the “theoretical” amplitude histogram matches the measured one.

In order to accurately reproduce the effects of the 4^th^-order Bessel filter used during the experiments and to allow for multi-state Markov models, we create the theoretical amplitude histogram by simulations. For each gating event, two random numbers are generated based on starting values of the rate constants in the model in Fig. 1d, one determining the sink state for a jump from the current source state, and one determining the dwell time in the source state before the jump occurs. After filtering the simulated time series of current, the histogram is calculated, and the base line noise is incorporated by a convolution of the amplitude histograms.

In repetitive runs, the rate constants of the model are varied by a Simplex algorithm^45^ until the amplitude histogram of the simulated time series shows a minimum deviation from that of the measured one. Details of this analysis in Kcv_NTS_ have been published previously^14^.

**Figure 2:**
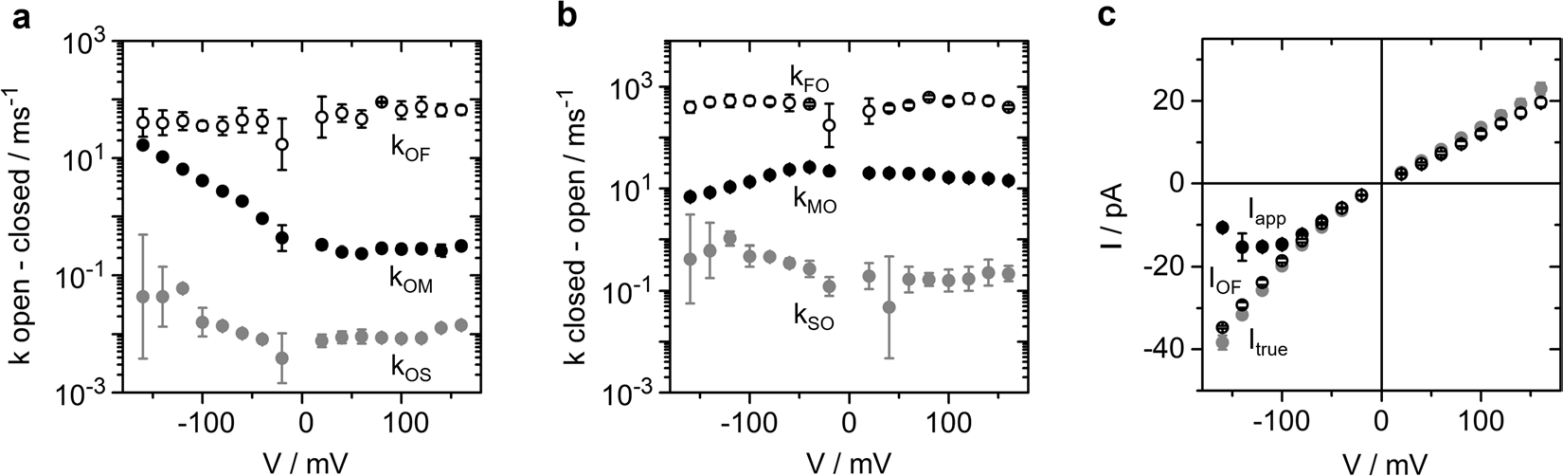
Results from fitting extended beta distributions to amplitude histograms like those in Fig. 1c in symmetrical KCl concentrations of 500 mM. **(a**,**b)** Rate constants of the gating processes in Fig. 1d (**c**) IV curves of *I*_*app*_ (black), *I*_*OF*_ (open circles) and *I*_*true*_ (grey). Mean and standard deviation from three different bilayers. If not visible, error bars are smaller than the symbols and below 25% for the rate constants (**a**,**b**) and below 1 pA for the currents (**c**), respectively.

Figure 2a,b shows the voltage dependence of the rate constants of the model in Fig. 1d in symmetrical solutions of 500 mM KCl. The rate constant of channel closure, *k*_*OM*_, related to the O-M transitions is the most voltage-dependent one. The rate constant of opening, *k*_*MO*_, is less voltage-dependent. The O-F gating is scarcely voltage-dependent. The error bars are large because the O-F rate constants are at the edge of the temporal resolution. The weak voltage dependence of *k*_*OS*_ and *k*_*SO*_ at negative potentials is not significant, because the large error bars indicate the uncertainty of their determination. The O-S gating is not investigated here.

The analysis by extended beta distributions discriminates three different currents (Fig. 2c). The apparent current, *I*_*app*_, is obtained directly from the time series after averaging over 10 adjacent sampling points. The true open-channel current *I*_*true*_ is the current, which would be measured with a fictional amplifier of infinite bandwidth and no noise. It can be revealed by fitting extended beta distributions to amplitude histograms. *I*_*OF*_ is the current for the case that O-M gating would be fully resolved, and the amplifier averages only over O-F gating (Supplementary Eq. S1). Calculating *I*_*app*_ by averaging over both the O-M and O-F gating results in the same value of *I*_*app*_ as obtained directly from the time series (Supplementary Eq. S2). We have verified the reliability of the determination of *I*_*OF*_ and *I*_*app*_ previously^14^. For the sake of comparison, the experimental data generated for the investigation here was supplemented by a few data from this method paper^14^. Details are given in the respective Figure legends.

### The O-M gating occurs in the selectivity filter

**Figure 3:**
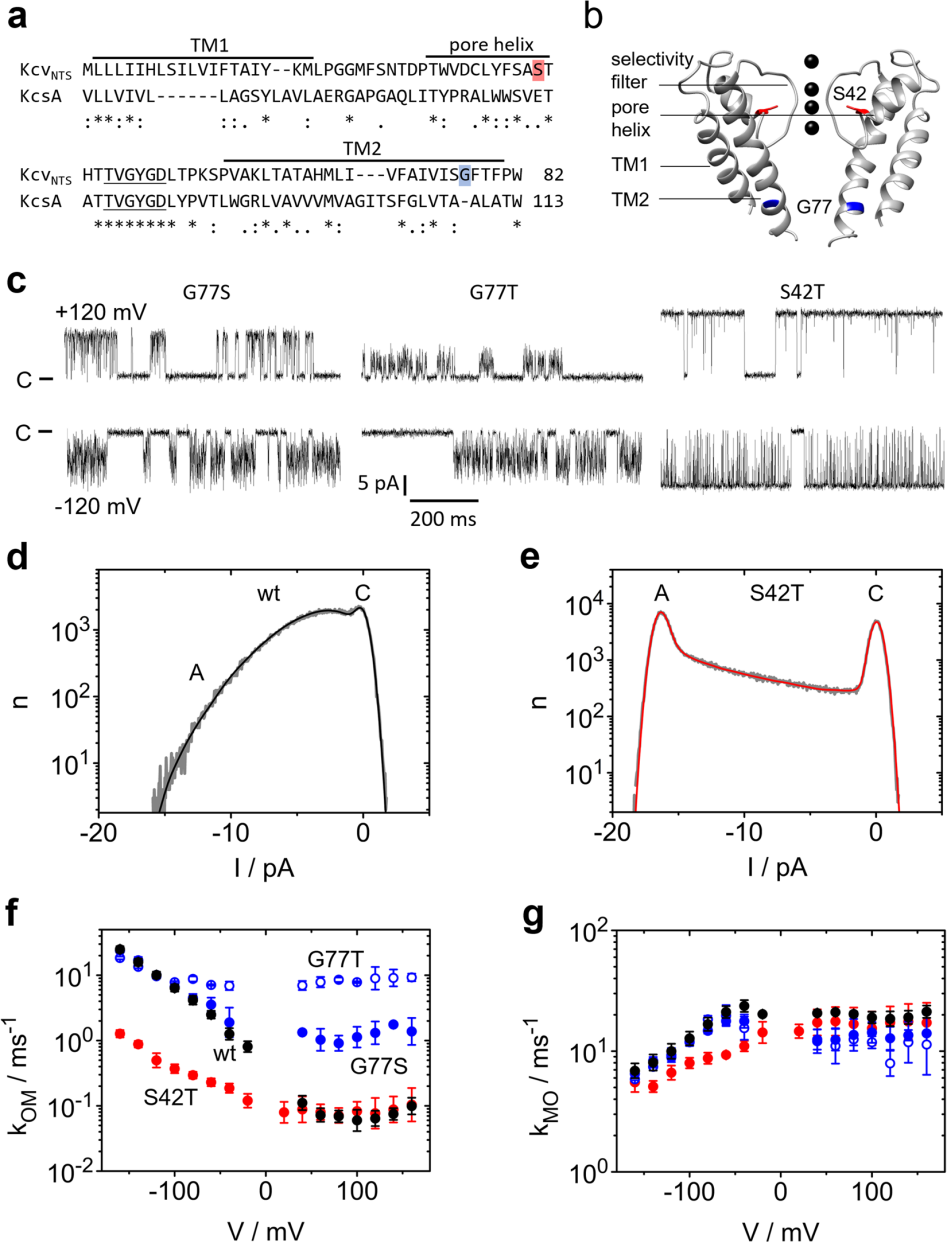
Mutational studies for the identification of the location of the two different processes found in O-M gating. **(a)** Sequence of Kcv_NTS_ as compared to KcsA. Asterisks indicate conserved residues, colons and periods strongly and weakly similar properties, respectively. **(b)** Structural model of Kcv_NTS_ constructed with Swissmodel^79^ with KirBac1.1 (PDB 1P7B^54^) as a template and drawn with UCSF Chimera^80^. The positions of mutations used in this study are indicated in red and blue, respectively. **(c)** Representative current traces of the mutants Kcv_NTS_ G77S, G77T, and S42T measured in symmetrical 100 mM KCl at −120 mV (top) and +120 mV (bottom). C labels the closed state. **(d**,**e)** Amplitude histograms (grey) of Kcv_NTS_ (**d**) and Kcv_NTS_ S42T (**e**) at −160 mV. The fit by the model in Fig. 1d with extended beta distributions^44^ is given in black or red. **(f**,**g)** Voltage dependence of the rate constants (**f**) *k*_*OM*_ and (**g**) *k*_*MO*_ of Kcv_NTS_ wt (black), Kcv_NTS_ S42T (red), Kcv_NTS_ G77S (blue) and G77T (blue open symbols). For comparison, the rate constants for Kcv_NTS_ wt and NTS G77S in (**f**) and (**g**) have been taken from a previous publication^14^. Numbers of experiments = 3 (6 for Kcv_NTS_ wt). If the standard deviations are smaller than the data points they are less than 25% scatter. All currents were recorded in symmetrical 100 mM KCl.

We have shown previously^14^ that in Kcv_NH_, a close homologue of Kcv_NTS_, the O-M gating is located in the selectivity filter. In Fig. 3, we show that this also holds for Kcv_NTS_. The structural model of Kcv_NTS_ suggests that the mutation S42T affects the anchoring of the selectivity filter (Fig. 3a,b). In KcsA, mutations of the corresponding residue (E71) have dramatic effects on the hydrogen-bond network surrounding the selectivity filter and on C-type inactivation^46^. In Kcv_NTS_ the mutation strongly influences the gating at negative voltages (compare Fig. 3c, right panel, with Fig. 1a) and thus the slope of the amplitude histogram between the closed peak and the open peak at negative voltages (Fig. 3d,e). This region is shaped by the O-M gating^14^. Analysis by extended beta distributions reveals that the S42T mutation drastically reduces the rate constant *k*_*OM*_ (Fig. 3f) and to a lesser extent *k*_*MO*_ (Fig. 3g).

In contrast, mutations at G77 (Fig. 3a,b) influence a newly detected inner gate^34^. At negative potentials, G77S and G77T do not have a significant effect on the time series (Fig. 3c) and on *k*_*OM*_ (Fig. 3f), where the curves merge into the slope, and on *k*_*MO*_ (Fig. 3g). In contrast, they have a significant effect on *k*_*OM*_ at positive voltages (Fig. 3c,f). The different action of the mutations at positive and negative voltages indicates that two different processes are involved in the O-M gating^14^. That one dominating at negative potentials is associated with the selectivity filter and that one at positive potentials with the inner gate^14^.

### The dependence of O-M gating and current on K^+^ concentration and voltage

The assignment of the O-M gating to the selectivity filter (Fig. 3) raises the question of whether its voltage dependence originates from changes in the interaction between the permeant ions and the filter. The effect of ion occupation in the selectivity filter has been considered by several authors, e.g.^6,10,47,48^, based on the effect of permeant ions on gating.

**Figure 4:**
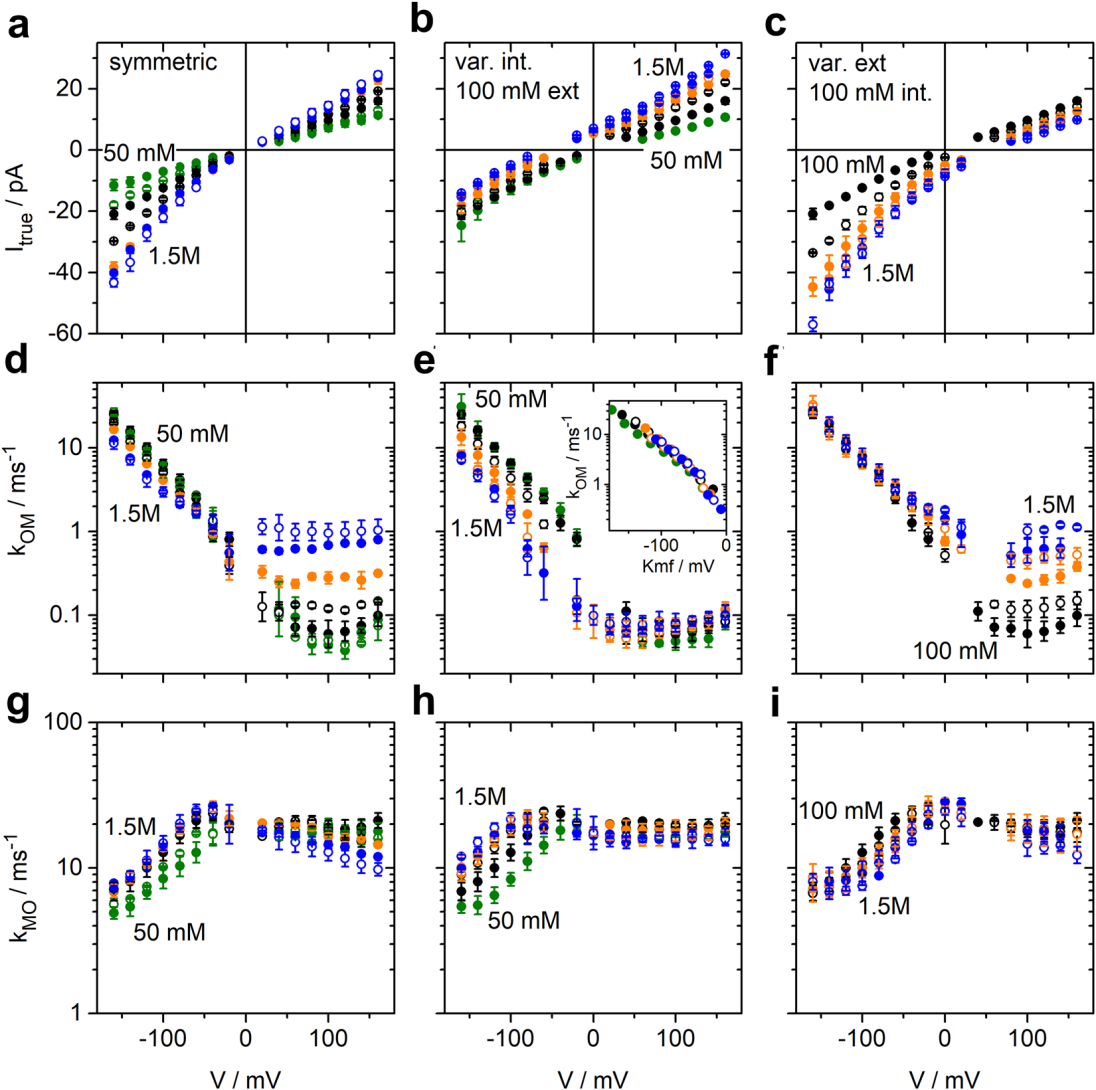
Results of fitting ca. 1000 amplitude histograms from Kcv_NTS_ like those in Figs 1c and 3d from recordings in symmetrical and asymmetrical KCl solutions. Voltage and concentration dependence of those three parameters are shown, which describe the O-M gating: **(a**,**b**,**c)** current *I*_*true*,_ **(d**,**e**,**f)** rate constant *k*_*OM*_ of channel closure, and **(g**,**h**,**i)** rate constant *k*_*MO*_ of channel opening. **(a**,**d**,**g)** Effect of symmetrical KCl concentrations ranging from 50 to 1500 mM. **(b**,**e**,**h)** Effect of internal K^+^: The external concentration was constant = 100 mM and internal concentration was varied. **(c**,**f**,**i)** Effect of external K^+^: internal KCl concentration was constant = 100 mM and external concentration was varied. 50 mM and 75 mM: green; 100 mM and 250 mM: black; 500 and 750 mM: orange; 1000 mM and 1500 mM: blue. The first concentration of a set of two with the same colour is given by closed circles, the second one by open circles. The inset in (**e**) shows the dependence on driving force *Kmf* = *V − E*_*K*_ for internal K^+^ concentrations, indicated by the congruence of the curves when plotted over *Kmf*. Mean and standard deviations are obtained from 3–4 independent experiments for each concentration (6 for 100 mM symmetric KCl). If the error bars are hidden by the symbols, they are smaller than 25% for the rate constants and below 1 pA for the currents. The rate constants and *I*_*true*_ for symmetrical 100 mM KCl, and the rate constants for symmetrical 1500 mM KCl have also been used in a previous publication^14^ to illustrate the extended beta distribution analysis.

Here, we employ a new approach to determine ion occupation in the filter from single-channel data. To this end, we determine the voltage dependence of the rate constants *k*_*OM*_ and *k*_*MO*_ of the O-M gating and of the open-channel current over a wide range of symmetrical and asymmetrical K^+^ concentrations, from 50 or 100 mM to 1500 mM (Fig. 4).

The IV curves (Fig. 4a–c) are about linear at symmetrical concentrations around 100 mM. This indicates that the voltage-sensitive ion translocation in the selectivity filter is not rate-limiting for the flux^49^. With increasing external K^+^ concentration, there is a weak exponential increase at negative voltages indicating that at high concentrations the voltage-sensitive translocation step starts to become rate limiting (Fig. 4c). This effect is much less pronounced at positive voltages for increasing internal concentrations (Fig. 4b) suggesting an asymmetry in the system. In symmetrical solutions (Fig. 4a), the effect of external concentrations dominates at negative voltages and the effect of internal concentrations at positive voltages (Fig. 4b).

The rate constant of channel closure, *k*_*OM*_, increases exponentially at negative voltages. It is strongly left-shifted with increasing internal K^+^ concentrations (Fig. 4e). Plotting *k*_*OM*_ versus the K^+^ motive force *Kmf* = *V* − *E*_*K*_, with *V* being the membrane voltage and *E*_*K*_ the reversal potential for K^+^, shows that *k*_*OM*_ depends on the *Kmf* under these conditions (Fig. 4e, inset). Below, this feature is discussed as a stronghold of our analysis as it is quite unexpected in terms of the relation between ion concentration at site S0 and the sensitivity of *k*_*OM*_ to internal K^+^ concentrations. In contrast, external K^+^ concentration has no effect (Fig. 4f) on *k*_*OM*_ at negative voltages. At positive voltages, *k*_*OM*_ is nearly voltage-independent, and the effect of K^+^ concentration is quite different from that at negative voltages as increasing internal K^+^ has no effect (Fig. 4e), whereas increasing external K^+^ has a strong effect (Fig. 4f).

The rate constant of channel opening, *k*_*MO*_, is also quite constant at positive voltages (Fig. 4g,h,i). The exponential dependence on negative voltages is opposite and less steep than that of *k*_*OM*_. The magnitude of the effect of K^+^ concentrations is similar for internal and external concentrations, but the effect is of opposite sign (Fig. 4h,i).

## Model-based IV curve analysis

### Models of ion permeation

**Figure 5:**
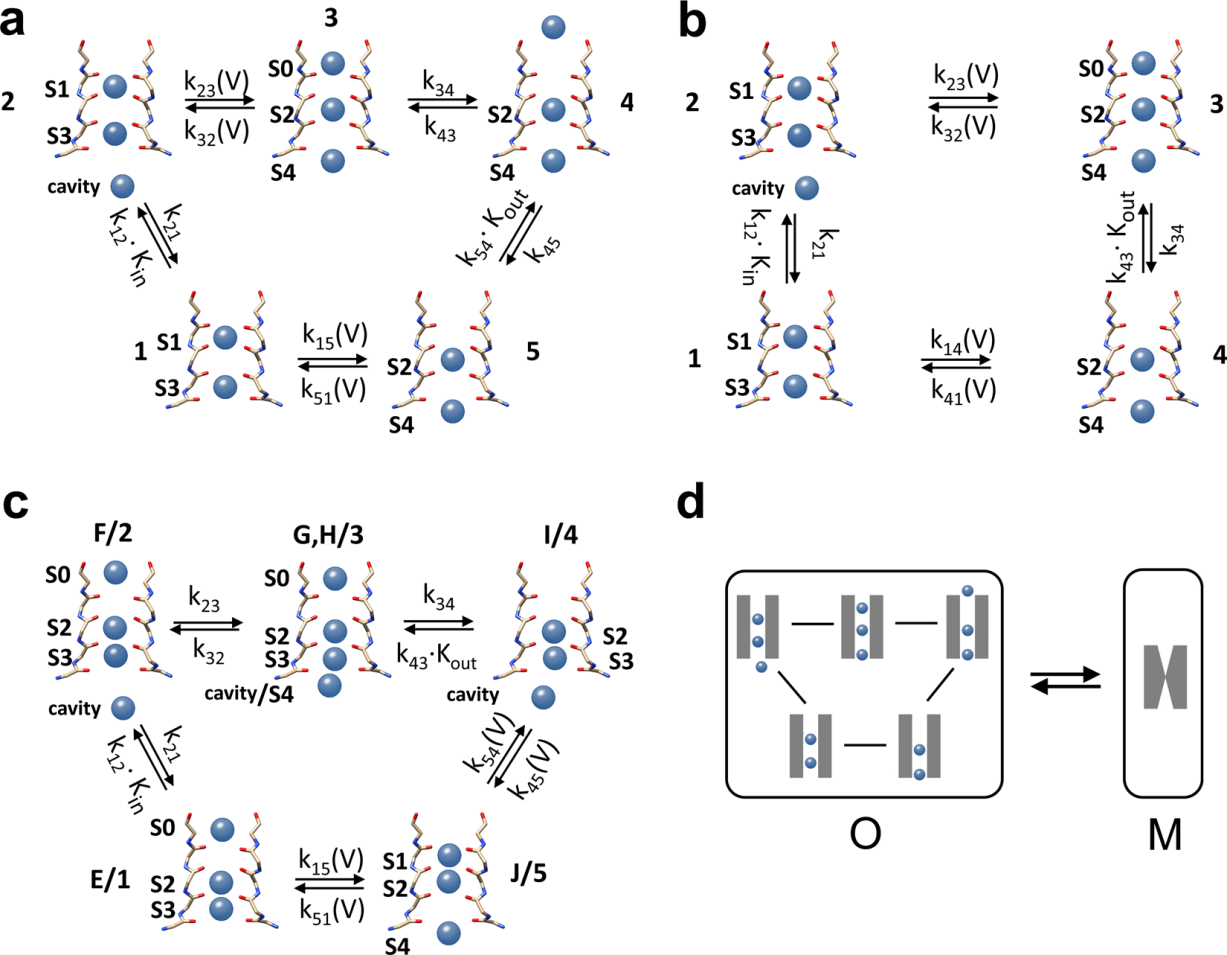
Different ion hopping models of the permeation process in the selectivity filter. **(a)** 5-state model of Roux^42^ **(b)** 4-state model generated by removing the outer binding site of the model in (**a**) **(c)** Direct knock-on model^43^. S0 to S4 label the binding sites of K^+^ ions in the selectivity filter, as defined in the papers mentioned above. The numbers in (**a**,**b**) label the ion configurations as used in Eqs 1–4. In (**c**), the labels “letter/number” indicate the correspondence between the labels of the configurations of Köpfer *et al*.^43^ and those used in the Eqs S15 to S17 in the Supplementary material. In the model in (**c**), two reactions from the original scheme^43^ are merged according to the theory of reserve factors^36^ because they are not affected separately by the experimental conditions. All models in (**a**–**c**) are permeation models representing the open state. This is illustrated in (**d**) showing that the O-M gating in Fig. 1d occurs between the kinetic closed state M and the kinetic open (conducting) state O, which includes all permeation states of one of the models in (**a**–**c**). The rate constants in those cycles are in the range of 10^7^ to 10^8^ / s, much faster than the rate constants of O-M gating. Thus, the occupation probabilities *P*_*m*_ of states *m* in Eqs 5 and 6 are voltage-dependent steady-state values. The dependence of the rate constants on K^+^ activity and voltage is indicated for each model as given by Eqs 2ab and 3 and in Supplementary Eqs S5–7, S15–17. The kinetic models are generated using the relevant information from the original papers.

Figure 3 shows that the O-M gating is physically located in the selectivity filter. Its sensitivity to voltage and K^+^ suggests that it reflects a distinct occupation of the filter by K^+^ ions. In Fig. 5a to c, three different models^42,43^ for ion hopping in the selectivity filter^50^ during the open state are used for setting up the equations for a quantitative analysis.

### The equations for the fit of the IV curves

We start the analysis with the model in Fig. 5a^42^ in order to investigate whether there is a set of rate constants of ion hopping, which can create the occupation probabilities *P*_*m*_ of the 5 states in the model. They determine the voltage dependence of measured current (IV curves) as well as that of the rate constant of channel closing *k*_*OM*_ for all K^+^ concentrations shown in Fig. 4. Here, only the central equations are listed. A full description is provided in Supplementary Eqs S3–S7.

The open channel current can be calculated from the rate constants *k*_*ij*_ between the states in Fig. 5a.

1 $$I=\mathrm{outward}\,\mathrm{current}-\mathrm{inward}\,\mathrm{current}=e\frac{{k}_{12}\cdot {k}_{23}\cdot {k}_{34}\cdot {k}_{45}\cdot {k}_{51}-{k}_{15}\cdot {k}_{54}\cdot {k}_{43}\cdot {k}_{32}\cdot {k}_{21}}{{D}_{1}^{C5}+{D}_{2}^{C5}+{D}_{3}^{C5}+{D}_{4}^{C5}+{D}_{5}^{C5}}$$

*e* is the unit charge, the numbers 1–5 represent the states in the ion-hopping model in Fig. 5a. The sum of the five 5 × 5 *D*-matrices in the denominator results in 100 products of 4 rate constants (Supplementary Eq. S4). As justified in Supplementary Note 1, *I*_*OF*_ (Fig. 2c, Supplementary Eq. S1) is used as effective current in Eq. 1 as it accounts for the averaging over the O-F gating occurring prior to an open-closed transition of the O-M gating.

The binding reactions *k*_12_ and *k*_54_ are proportional to the ion activities *a*_*K*_ (instead of concentrations, see Supplementary Table S1)

2ab $${k}_{12}={k}_{12,1}\cdot {a}_{Kin},{k}_{54}={k}_{54,1}\cdot {a}_{Kout}$$

The last index “1” labels the rate constants for *a*_*K*_ = 1 mM.

The two most voltage-dependent reactions are those where two ions move simultaneously through the centre of the selectivity filter. The effect of voltage on the other reactions is expected to be small, since 80% of the voltage is assumed to drop over the selectivity filter^51–53^. Assuming an Eyring barrier at the electrical position *s*_*ij*_ (*i*,*j* = 2,3 and 5,1) leads to

3 $${k}_{ij}={k}_{ij,0}\cdot \exp (\frac{{s}_{ij}V}{{V}_{ij}})$$

*V*_*ij*_ is the characteristic voltage (causing an *e*-fold increase) of the respective state transition. The index “0” labels the rate constants at 0 mV. Details are given by Supplementary Eqs S5–S7.

### The equations for the fit of the *k*_*OM*_(V) curves

The modulation of the forces between ions and protein occurs on the time scale of permeation, i.e. around 10 ns. In contrast, gating is much slower. Thus, the effect of ion occupation on gating results from a long-term integration over those forces and is related to the steady-state ion occupation probabilities *P*_*m*_ (*m* = 1 to 5) of the model in Fig. 5a. They are calculated by means of the *D*-matrices, which occur in Eq. 1 and are described in Supplementary Eq. S4.

4 $${P}_{m}=\frac{{D}_{m}^{C5}}{{D}_{1}^{C5}+{D}_{2}^{C5}+{D}_{3}^{C5}+{D}_{4}^{C5}+{D}_{5}^{C5}}$$

An enzyme is a protein where an allosteric site can influence the kinetics of the active site. In analogy to this, an ion channel can be seen as a protein, where an “allosteric site” can influence the kinetics of a distant gate. Considering what is known about the mechanism of the action of an allosteric site on enzyme activity, we have to consider two different mechanisms, which may mediate the allosteric effect of *P*_*m*_ on gating. As discussed in detail below, this can be achieved by a change in conformation or a change in flexibility^19^. Thus, we must distinguish between two basically different possible mechanisms.

1. The forces between ions and carbonyl groups cause a conformational change at the site of the gate. In this case, we expect that *k*_*OM*_ is proportional to one or more of the occupation probabilities *P*_*m*_ *o*f state *m* (*m* = 1 to 5) in the model of Fig. 5a.
2. The forces between ions and selectivity filter cause a change in the flexibility of the protein. As discussed in detail below, such an effect is well-known from the allostery of enzymes and is also considered for ion channels. In that case, *k*_*OM*_ would be related to 1/*P*_*m*_ (details see Discussion and Eq. 8, below).

Thus, we start with a test of the following relationships

5ab $${k}_{OM}=w\cdot {P}_{m}\,\mathrm{or}\,{k}_{OM}=w\cdot 1/{P}_{m}$$

with *w* being a scaling factor independent of voltage and K^+^ concentration, and *m* = 1 to 5.

### Identification of the voltage-sensing binding site(s) by a global fit of the IV curves and *k*_*OM*_(V)

We search for a set of rate constants of the ion hopping model in Fig. 5a, which can create the measured IV curves as well as the measured dependence of *k*_*OM*_ (related to appropriate ion distributions *P*_*i*_, Eqs 4,5) on negative voltage and K^+^ concentration.

This is achieved by a global fit of the IV curves in Fig. 4a to c and the *k*_*OM*_(*V*) curves in Fig. 4d to f including all 36 curves (18 for IV and 18 for *k*_*OM*_(*V*)) obtained with different K^+^ concentrations. For the IV curves, Eq. 1 is used. For *k*_*OM*_(*V*), one of the 10 equations given by Eq. 5a,b is employed (for *m* = 1 to 5). However, to give about equal weight in the fitting routine to the IV curves and the rate constant ln(*k*_*OM*_) is fitted. The global fit implies that for both equations (Eq. 1 and one of Eq. 5a,b) the identical set of free parameters is used. These are the rate constants *k*_*ij*_ of the ion hopping model in Fig. 5a, the parameters *s*_51_, *s*_23_, *V*_51_ and *V*_23_ of the Eyring barriers (Eq. 3) and the scaling factor *w* in Eq. 5.

The resulting error sums from the global fits provide a measure of how well each one of the 10 options (Eq. 5a,b) is in agreement with the data (Table 1). The lowest error sum and thus the best fit is achieved for the global fit of the IV curve together with *k*_*OM*_ = w*1/*P*_3_ (Supplementary Fig. S1). Other ion occupancies than 1/*P*_3_ result in significantly worse fits (Table 1 and Supplementary Fig. S2). Interestingly, *P*_3_ is the only occupation pattern in the conduction cycle with three ions in the filter and in which the outermost binding site S0 is occupied (Fig. 5a). In other words, the probability of channel closing is increased when the number of ions in the filter decreases and S0 becomes empty. The involvement of S0 is not surprising because negative voltage pulls the ions away from S0.

**Table 1:** Errors sums of the global fits of the IV curves with the model in Fig. 5a and of ln (*k*_*OM*_) with the theoretical *k*_*OM*_ curve calculated from a selected pattern of ion distribution (Eqs 5 and 6) as given in the first row.

Partner of IV	*P*_*1*_	*P*_*2*_	*P*_*3*_	*P*_*4*_	*P*_*5*_	*1/P*_*1*_	*1/P*_*2*_	*1/P*_*3*_	*1/P*_*4*_	*1/P*_*5*_	*P*_*4*_ */P*_*3*_
Error sum of best fit	1056	1425	884	655	673	706	550	**452**	662	2117	**282**

**Figure 6:**
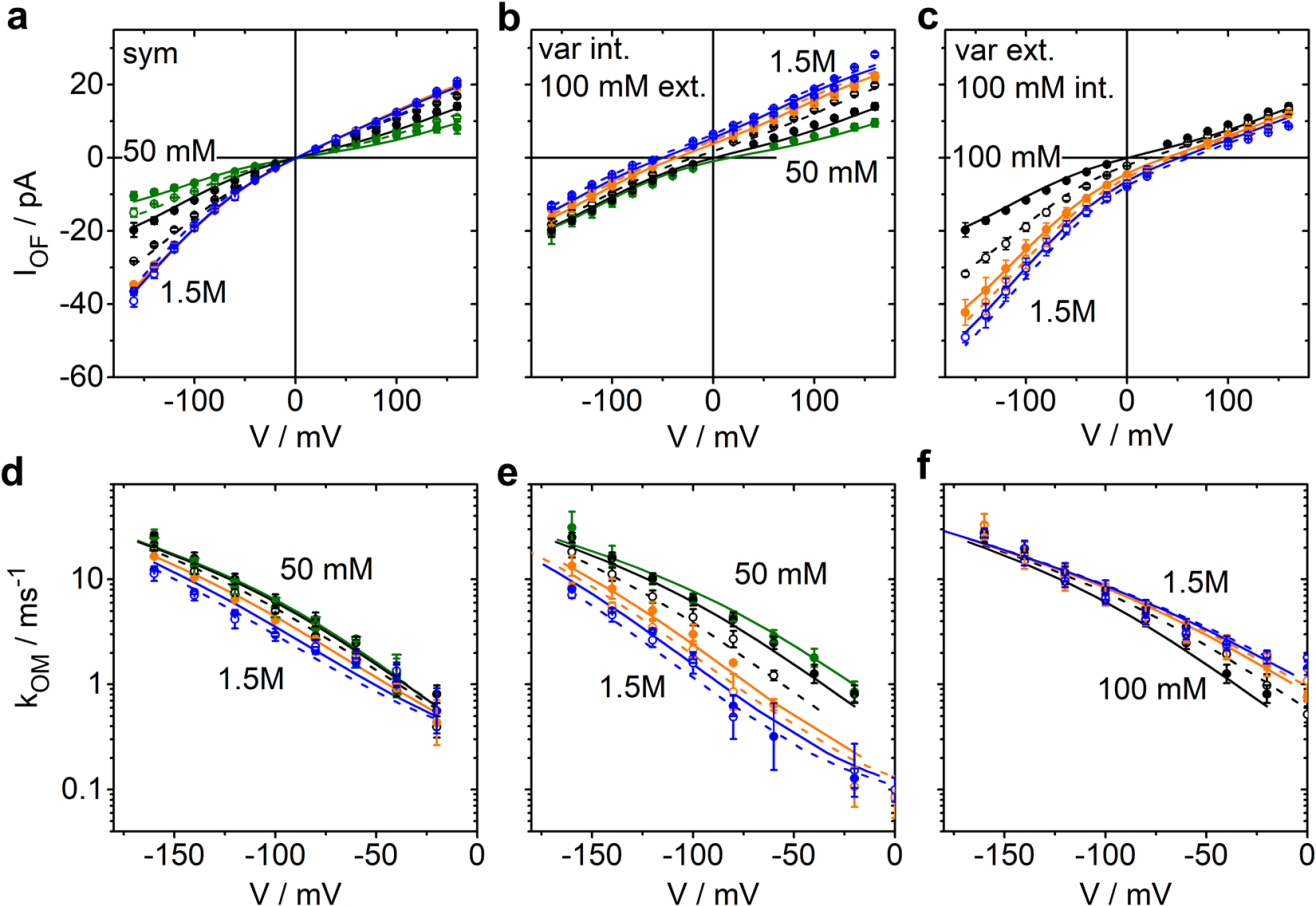
Global fits **(a–c)** of the IV curves by Eq. 1 and (**d–f**) of ln(*k*_*OM*_) = ln(*wP*_4_*/P*_3_) (only at negative voltages as justified by Fig. 3f,g) by Eq. 6 for different **(a**,**d)** symmetrical, **(b**,**e)** internal and **(c**,**f)** external K^+^ concentrations. All curves have been fitted with the same set of parameters as given in the last column of Supplementary Table S2. The concentrations are indicated by the colours: 50 mM and 75 mM: green; 100 mM and 250 mM: black; 500 and 750 mM: orange; 1000 mM and 1500 mM: blue. Circles represent the data points, lines the theoretical curve obtained from the global fit. The first concentration of a set of two with the same colour is given by closed circles and a continuous line, the second one by open circles and a dashed line. Error bars results from three experiments (6 for symmetrical 100 mM). In the case of the IV curves, the error bars are so small (less than 1 pA) that they are mostly hidden behind the symbols. The current and rate constants for 100 mM, the current for 1000 mM and the rate constants for 1500 mM have been taken from a previous publication^14^.

The fit, which uses *k*_*OM*_ = w*1/*P*_3_, matches the measured IV curves quite well and also the voltage dependence of *k*_*OM*_ (Supplementary Fig. S1). However, the fit overestimates the concentration dependence of *k*_*OM*_ on symmetrical KCl. The concentration dependence and even the steepness of the voltage dependence of the fit can considerably be improved (Table 1) by incorporation of *P*_4_, the neighbouring state of *P*_3_, by means of the equation

6 $${k}_{OM}=w\frac{{P}_{4}}{{P}_{3}}$$

with *w* also here being a constant scaling factor. The parameters of the global fits of the IV curves and ln(*k*_*OM*_) = ln(*w P*_4_*/P*_3_) are provided in Supplementary Table S2. The IV curves are well fitted, and good fits of the concentration dependence of *k*_*OM*_ are obtained (Fig. 6d–f) except for some minor deviations at extreme negative voltages where the characteristics of the amplitude histograms become less pronounced.

In the 5-state ion hopping model of Fig. 5a, the occupation patterns of states 3 and 4 are very similar. The only difference is that the ion, which occupies S0 in state 3 (*P*_3_), is in the outer pore mouth outside of S0 in state 4 (*P*_4_). States 3 and 4 do not occur simultaneously. However, at a current of 16 pA one transport cycle takes 10 ns. Thus, on the time scale of *k*_*OM*_, the protein feels the averaged effects of *P*_3_ and *P*_4_ simultaneously. The qualitative mechanistic interpretation of Eq. 6 is: Three ions in the filter including S0 (*P*_3_) stabilize the open state via an increase in rigidity (decrease in flexibility), and an ion in the outer pore mouth (*P*_4_) destabilizes the open state via a conformational change or a change in force (Eq. 8, below). Evidence for the reliability of the fitting routines is provided in Supplementary Note 2.

### Test of alternative models of ion permeation

We tested, whether the model can fit the experimental data when the binding site for the outer ion (state 4 of Fig. 5a) is omitted. For fits based on the resulting 4-state model in Fig. 5b, again all assignments *k*_*OM*_(*V*) to *P*_*m*_ or 1*/P*_*m*_ (*m* = 1 to 4) were tested in global fits with the IV curves. Eqs 2 and 3 were also used here. Similar to the 5-state model in Fig. 5a used above, the voltage dependence could only be reproduced by assuming *k*_*OM*_ = w*1/*P*_3_ (Supplementary Fig. S3). However, this model failed to provide the dependence on K^+^ concentration. In contrast to the model in Fig. 5a, this could not be repaired by providing a partner for 1/*P*_3_ as in the case of *k*_*OM*_ = *w P*_3_/*P*_4_ in the 5-state model in Fig. 5a. This indicates the necessity of state 4 in Fig. 5a.

In the case of the “hard knock-on” model^43^, an appropriate assignment of Eqs 2 and 3 (Supplementary Eqs S15–S17) led to a 5-state model with a different topology as in Fig. 5a. This model (Fig. 5c) was unable to reproduce the voltage dependence of *k*_*OM*_. Again, all assignments of *k*_*OM*_ to *P*_*m*_ or 1/*P*_*m*_ were tested. The “best” fit is shown in Supplementary Fig. S4.

### The understanding of an a priori non-intuitive action of external K^+^ concentration

**Figure 7:**
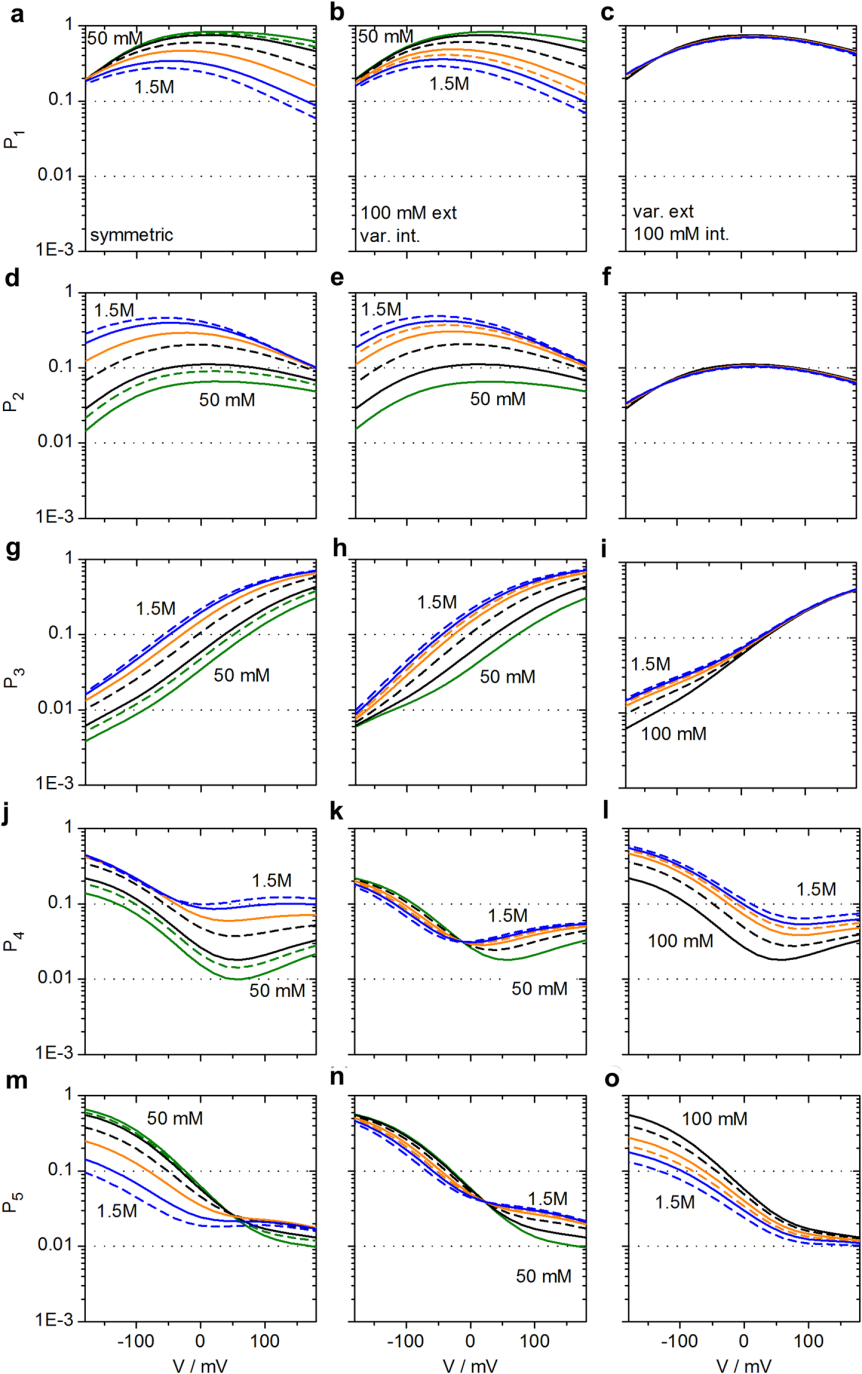
Occupation probabilities *P*_1_ to *P*_5_ of the states of the model in Fig. 5a for symmetrical solutions of K^+^ **(a**,**d**,**g**,**j**,**m)**, 100 mM external and internal concentration varied **(b**,**e**,**h**,**k**,**n)** and 100 mM internal and external concentration varied **(c**,**f**,**i**,**l**,**o)**. The *P*_*m*_ are calculated from the rate constants *k*_*ij*_ of the transitions between state *i* and *j* (Eq. 4, Fig. 5a and Table S2) as obtained from global fitting of the IV curves (Eq. 1) and *k*_*OM*_ = *w P*_4_*/P*_3_ (Eq. 6) as shown in Fig. 6. The colours give the K^+^ concentrations: green 50 and 75 mM, black 100 and 250 mM, orange 500 mM and 750 mM, blue 1000 and 1500 mM. In each pair with the same colour, the dashed lines give the higher concentration.

Unexpected is the finding in Fig. 4f that *k*_*OM*_ at negative voltages is not sensitive to external K^+^ concentration. In the light of the above mechanism of ion depletion around S0 it would be assumed that increasing external K^+^ concentration would increase *P*_3_ and thus decrease *k*_*OM*_. Inspection of *P*_4_ in Fig. 7 provides an explanation for the conundrum. At −160 mV, the effects of K^+^ concentration on *P*_3_ (ion at S0) and *P*_4_ (ion outside of S0) are similar (Fig. 7i and l), thus compensating each other in the ratio *P*_3_/*P*_4_. This compensation levels off when the voltage approaches 0 mV (Fig. 6f), thus reproducing the experimental finding in Fig. 4f. In symmetrical solutions, there is a partial compensation (Figs 6d and 7g,j). The numerical details of these effects are given in Supplementary Eq. S14.

### Changing internal K^+^ concentration results in a dependence of *k*_*OM*_ on driving force but does not allow a straight-forward identification of the location of the sensor

In contrast to external K^+^ concentrations discussed above, internal K^+^ concentrations do not have similar effects on *P*_4_ and *P*_3_ (Fig. 7h,k). Thus, the K^+^ effect on 1/*P*_3_ (Fig. 7h) becomes dominant as it is not compensated (Fig. 4e). The effect of varying internal K^+^ concentration can be accounted for by the *Kmf*, the sum between (weighted) electrical potential, *V* and Nernst potential (*E*_*K*_) (inset of Fig. 4e). The numerical origin for the dependence on *Kmf* for internal concentrations is given in Supplementary Eqs S8–S13, resulting in an equation of the form

7 $$\frac{{P}_{4}}{{P}_{3}}\approx \frac{cons{t}_{2}}{cons{t}_{1}\cdot \exp [Km{f}_{1}]+\exp [Km{f}_{2}]}\cdot $$

*Kmf*_1_ and *Kmf*_2_ account for the fact that different fractions of voltage are felt by the involved reactions.

This result demonstrates that an effect of internal concentration on *k*_*OM*_ cannot automatically be taken as evidence for an internal localization of the sensor for *k*_*OM*_. The analysis reveals that high internal concentrations hinder the withdrawal of ions from S0, and that this is not compensated by *P*_4_.

## Discussion

### The adequate permeation model for Kcv_NTS_ and identification of the ion occupation acting as a sensor for voltage-dependent O-M gating

The present study demonstrates the benefits of combining information from single-channel recordings and structural models for ion transport through the selectivity filter of a viral K^+^ channel.

From crystal structure analysis, it is known that the permeating ions occupy distinct binding sites in the selectivity filter^23,51,54,55^. This implies that transport occurs via hopping of the ions from one binding site to the next^50^. This process can be described by continuum or discrete approaches. For the continuum approaches, electro diffusion as described by the Poisson-Nernst-Planck equation is suggested^56^. Others^57^ argue that this approach has its limitations like the failure to distinguish between Na^+^ and K^+^ ions. They suggest to account for protein-ion interaction by incorporating changes in the dielectric constant via the Born equation. In the discrete approaches, the rate constants of ion hopping are determined from molecular dynamics (MD) simulations. Here, the problems are in the assumption of the correct simulation parameters leading to different permeation models as in Fig. 5a,c.

However, the individual problems of these approaches are not a concern, but an incentive for our investigation. We want to provide numerical parameters obtained from experiments, namely the ion occupation from the measured IV curves as provided by the fits described above. They can be used in subsequent approaches as a test of the predictions of the theoretical models by interactive mutual improvements of experimental approaches as provided here and computational approaches by Born-Poisson-Nernst-Planck or MD simulations.

For Kcv_NTS_, the best fits in a comparative model-based analysis are obtained with the 5-state model of ion hopping^42^ (Fig. 5a). The two alternative models (Fig. 5b,c) were not able to provide an adequate fit of the experimental data in the same frame of simple relationships. Of course, it can be argued that a more complex function of a weighted distribution of more than two states could also lead to successful fits in the case of the other models. However, we apply Occam’s Razor, i.e., giving preference to that model, which gives the simplest relationship. A stronghold of the model is the correct prediction of the non-intuitive asymmetrical action of internal and external K^+^ concentration.

The voltage dependence of the rate constant *k*_*OM*_, which is responsible for the apparent negative slope conductance in this channel, can fully be explained by the reduction of the number of K^+^ ions in the selectivity filter by the depletion of the binding site S0. This depletion on the external side of the selectivity filter is the result of an increasing inward current *I*_*OF*_ where negative voltage pulls the ions away from S0.

### The mediator between sensor and gate: changes in conformation or flexibility?

A peculiarity in the fitting with the 5-state model^42^ and also the 4-state model is that the best results are obtained with a dependence of *k*_*OM*_ on 1/*P*_3_. It may be considered that the effect of ion depletion at S0 could also be obtained by a more straight-forward term like 1-*P*_3_ in the global fits. However, in contrast to 1/*P*_3_, 1-*P*_3_ cannot account for the whole range of experimental values of *k*_*OM*_ (Fig. 6), which change with negative voltage over two orders of magnitude. Thus, we have to think of a likely molecular mechanism, which is able to account for this unusual relationship.

A possible explanation comes from the comparison of enzymes and ion channels. Both types of proteins can have allosteric sites, where a modulator (ligand) binds, and a distant active (orthosteric) site. The mechanisms of coupling between these sites are well-understood in enzymes since they are easier to crystalize. Thus, they provide suggestions for the phenomena investigated here. The carbonyl groups of the selectivity filter can be seen as the “allosteric site” in which the complexed ion acts as an “allosteric modulator”^23,24,29,31^. The active site is a still unknown gate.

Following this analogy, an ion could modulate the performance of the protein via two different mechanisms: a change in conformation or a change in flexibility. A modulatory impact of flexibility on protein function is well known from allostery in enzymes and documented by far infrared (FIR) spectroscopy^58^, change in melting temperature^59^, neutron scattering^60^, crystal structure B-factors^61^ or from computational studies^62^. The change in flexibility seems to play a more important role than usually anticipated. The modulation of an active site by an allosteric site in these systems is not only mediated by long-range conformational changes, but also by changes in the flexibility without obvious conformational changes^63^. Panjkovich and Daura^64^ performed normal-mode analysis and observed significant changes in protein flexibility upon allosteric ligand binding in 70% of the investigated cases.

This general phenomenon of protein flexibility seems to be relevant also in ion channels. For example, the snug fit model for selectivity^55^ has been replaced by models considering the flexibility of the selectivity filter^65^. Further examples for a role of protein flexibility in relation to structure and function of ion channels are also known for gramicidine A^19,21,66^, K^+^ channels^19,20,67,68^, NavAb^22^ or a CNG chimera^69^. Also in the model K^+^ channel KcsA, infrared (IR) spectroscopy has demonstrated that exchanging Na^+^ for K^+^ in the filter changes the vibrational modes of the ion-coordinating carbonyls^70^.

In the context of the present analysis we can speculate that a change in flexibility (or stiffness K) can cause a kink or an angle of a turn Δ*x* at the O-M gate according to the law of a spring^19^.

8 $${\rm{\Delta }}x=\frac{F}{K}$$

Thus, if the ion at site S0 increases stiffness, *P*_3_ is in the denominator. *P*_4_ is a possible candidate for modulating the force *F*.

*P*_4_ could also act via a change in flexibility since ligand binding can do both, increase or decrease flexibility. Such a behaviour was found in the activation in rhodopsin-like G protein-coupled receptors. It can constrain the movement of the retinal β–ionone ring (corresponding to *P*_4_) while at the same time increasing the flexibility in the ligand chain (corresponding to 1/*P*_3_)^71^.

In KcsA, the mutation E71A (corresponding to S42T in Kcv_NTS_, Fig. 3a,b) causes a profound rearrangement of the hydrogen-bond network surrounding the selectivity filter^46^. For the study of the detailed mechanism of the hypothetical connection between the sensor S0 and the still unknown O-M gate in Kcv_NTS_, this finding may serve as a first incitement to check whether such an effect is also involved in Kcv_NTS_.

## Conclusion

The model-based IV curve analysis has identified the pattern of ion occupation in the selectivity filter responsible for O-M channel closure at negative voltages in the viral K^+^ channel Kcv_NTS_. The confidence in this approach is supported by a correct and detailed description of the voltage- and K^+^-dependent sub-ms gating in the selectivity filter. The same analysis performed for other channels and other kinds of gating like C-type inactivation will provide an answer to the question of whether this mechanism holds also for other channels and which types of gating are correlated with which patterns of ion distribution in the selectivity filter. The present analysis does not answer the question of the nature of the gate. However, a combination of model-based IV curve analysis with measurements of protein flexibility supported by mutational studies, which address the putative link between the sensor and the gate, are expected to solve this problem. Furthermore, the rate constants reach a range, which in near future may by accessible to determination of the rate constants by computational methods.

## Methods

### *In vitro* protein expression and purification

Kcv_NTS_ is a member of the ATCV subfamily of the Kcv family^33,72^. The virus encoding for the channel was originally isolated from an alkaline lake in Nebraska^73^. The proteins were expressed *in vitro* and purified as described previously^34^. Briefly, the gene of Kcv_NTS_ was cloned into a pEXP5-CT/TOPO®-vector (Invitrogen, Karlsbad, CA, USA), the fusion of the His tag coded for in the plasmid was prevented by inserting a stop codon. Mutations were introduced by site-directed mutagenesis, following the QuickChange method^74^. All mutants were sequenced.

*In vitro* expression of the channel protein was performed with the MembraneMax^TM^ *HN* Protein Expression Kit (Invitrogen) following the manufacturer’s instructions. During the expression procedure, the Kcv_NTS_ proteins were directly embedded into nanolipoproteins (NLPs)^75^. The NLPs contained multiple His-tags, allowing the purification of the native Kcv_NTS_ proteins by metal chelate affinity chromatography. Purification was done on a 0.2 mL HisPur^TM^ Ni-NTA spin column (ThermoFisher Scientific, Waltham, MA, USA). Divergent from the manufacturer’s instructions, the column was washed three times with two resin-bed volumes of 20 mM imidazole to remove unspecific binders. The Kcv_NTS_-containing NLPs were then eluted in three fractions (200 µL each) with 250 mM imidazole. Neither the washing nor the elution solutions contained salts; this improves the reconstitution efficiency into the bilayer^76^.

### Lipid bilayer experiments

Planar lipid bilayer experiments were performed at room temperature (20–25 °C) with a vertical bilayer setup (IonoVation, Osnabrück, Germany). The recording chambers were prepared as described previously^72^, and 1,2-diphytanoyl-*sn*-glycero-3-phosphocholine (DPhPC, Avanti Polar Lipids, Alabaster, AL, USA) bilayers were formed using the pseudo painting/air bubble technique^77^. For reconstitution of the channel protein, one of the elution fractions was diluted in 250 mM imidazole solution by a factor of 1000 to 100,000, and a small amount (1–3 µL) of the diluted NLP/Kcv_NTS_-conjugates was added directly below the bilayer in the *trans* compartment with a bent Hamilton syringe.

After verifying the incorporation of a single channel in the lipid bilayer by short voltage pulses, constant voltages between +160 mV and −160 mV in steps of 20 mV were applied for 1 to 5 minutes. Both compartments of the bilayer chamber were connected with Ag/AgCl electrodes to the headstage of a patch-clamp amplifier (L/M-EPC-7, List-Medical, Darmstadt, Germany). Membrane potentials were applied to the *cis* compartment, the *trans* compartment was grounded. Experiments with asymmetrical KCl concentrations were performed with 3 M KCl agar bridges. K^+^ concentration was changed by replacing an appropriate amount of the solution in the recording chamber by a 500 mM, 1.5 M or 3 M KCl stock solution and thorough mixing. All solutions and stock solutions contained 10 mM HEPES, pH was adjusted to 7.0 with KOH. The asymmetric nature of the apparent IV curves (Fig. 2c) of Kcv channels^33^ allows the identification of the orientation of the channel in the bilayer. In all graphs, positive currents corresponded to outward currents in the *in vivo* situation. Currents were filtered with a 1-kHz 4-pole Bessel filter and digitized with a sampling frequency of 5 kHz (LIH 1600, HEKA Elektronik, Lambrecht, Germany).

### Correction of electrode potentials

During the long experiments (up to 10 hours for the recording of steady-state IV curves in up to 10 different KCl concentrations), the electrode potential could drift by about 2 to 20 mV (the average of absolute values was 4 mV) despite the use of agar bridges. Since K^+^ is the only cation present in the solution, the reversal potential could be corrected retroactively. To this end, the measured apparent reversal potential of the IV curve of *I*_*OF*_ (definition in Supplementary Eq. S1) was determined by a 3^rd^-order polynomial fit. The rate constants and currents for each individual voltage protocol were then shifted along the V-axis accordingly. In cases where several experiments were averaged, the data points were interpolated with a cubic spline function before averaging. Changes in the KCl concentrations did not occur because of the large volume of the chambers (2.5 mL).

### Determination of *I*_*true*_ and the rate constants of fast gating from extended beta distribution analysis

Gating is strongly attenuated if its rate constants are higher than the low-pass filter frequency of the set-up. Under these circumstances, neither the individual gating transitions, nor the true open channel current, *I*_*true*_, can be directly observed. Fortunately, gating causes “excess noise”^44,78^, which causes broadened, non-Gaussian peaks in the amplitude histograms (see Figs 1c and 3d,e). From the deviations of the measured amplitude histograms from that one resulting from the Gaussian baseline noise, the hidden gating processes can be resolved by extended beta distribution analysis^44^ using our lab-made program “*bownhill*”. The analysis is based on the simulation of theoretical time series from a Markov model of gating (Fig. 1d), and thus it can include without any simplifying assumptions all peculiarities of the individual experiments, like set-up noise, characteristics of the higher order low-pass filter, and adequate multi-state Markov model of gating^44^. Details of the application of this analysis to gating in Kcv_NTS_ have been described previously^14^. The program *bownhill* can be downloaded from http://www.bio.tu-darmstadt.de/ag/professuren/indraschroeder/software.en.jsp.

The datasets generated and/or analysed during the current study are available from the corresponding author on reasonable request.

## Electronic supplementary material

Supplemental Material
