# Supplementary material for: Site-specific ion occupation in the selectivity filter causes voltage-dependent gating in a viral K+ channel
Source: Sci Rep. 2018 Jul 10;8:10406. doi: 10.1038/s41598-018-28751-w (PMC6039446; doi:10.1038/s41598-018-28751-w)
Supplement: Supplementary file 1 — Supplemental Material [file 41598_2018_28751_MOESM1_ESM.pdf]

Supplementary Information to

Site-specific ion occupation in the selectivity filter causes voltage-dependent gating in a viral K+ channel

O. Rauh, U.P. Hansen, D.D. Scheub, G. Thiel and I. Schroeder

Supplementary Equations 1 and 2

$$I_{OF} = I_{true} \frac{k_{FO}}{k_{FO} + k_{OF}} \qquad \text{(S1)}$$

$$I_{app} = I_{true} \frac{k_{FO}}{k_{FO} + k_{OF}} \cdot \frac{k_{MO}}{k_{MO} + k_{OM}} = I_{OF} \frac{k_{MO}}{k_{MO} + k_{OM}} \qquad \text{(S2)}$$

Supplementary Equations 3 to 7: Detailed equations for the fit of the IV curves

The equations are given in detail for the model in Fig. 5a[1]. This cyclic model has five different states of ion occupancy. The open channel current can be calculated from the rate constants k_{ij} between these states. (The indices of the rate constants of the ion hopping models are given as numbers or by *i,j* if not specified, those of the gating process (Fig. 1d) by letters excluding *i,j*). The equation for fitting measured IV curves has been set up with the so-called "arrow scheme"[2]. This algorithm makes the inherent pattern of rate equations obvious and thus enables an easy generation of flux equations:

$$I = \text{outward current - inward current} = e \frac{\overset{1\;\;2\;\;3\;\;4\;\;5\;\;1}{[\rightarrow\rightarrow\rightarrow\rightarrow\rightarrow]} - \overset{1\;\;2\;\;3\;\;4\;\;5\;\;1}{[\leftarrow\leftarrow\leftarrow\leftarrow\leftarrow]}}{D_1^{C5} + D_2^{C5} + D_3^{C5} + D_4^{C5} + D_5^{C5}} \qquad \text{(S3)}$$

e is the unit charge, the numbers 1-5 represent the states in the ion-hopping model (Fig. 5a). An arrow between the numbers *i* and *j* stands for the rate constant k_{ij} or k_{ji} depending on the direction of the arrow. Each row in square brackets gives the product of the rate constants. The matrices D_i^{C5} (C5 means "cyclic 5-state model") are defined as follows

$$D_1^{C5} = \overset{1\;\;2\;\;3\;\;4\;\;5\;\;1}{\begin{bmatrix} & \rightarrow & \rightarrow & \rightarrow & \rightarrow \\ \leftarrow & & \rightarrow & \rightarrow & \rightarrow \\ \leftarrow & \leftarrow & & \rightarrow & \rightarrow \\ \leftarrow & \leftarrow & \leftarrow & & \rightarrow \\ \leftarrow & \leftarrow & \leftarrow & \leftarrow & \end{bmatrix}} = \sum_{m=1}^{5} \prod_{i=2}^{m} k_{i,i-1} \prod_{j=m+1}^{5} k_{j,j+1} \qquad \text{(S4)}$$

The rate constants in a row represented by an arrow are multiplied, and the products of each row are added. The indices are cyclic, i.e., the index following 5 is not 6, but 1. For the matrices D_2^{C5} to D_5^{C5}, the indices are rotated starting with the subscript of the matrix. For details, see ref.[2]. Ion activities a_K (instead of concentrations, see Supplementary Table S1) are inserted into the binding reactions by

$$k_{12} = k_{12,1} \cdot a_{Kin} \qquad \text{(S5a)}$$

$$k_{54} = k_{54,1} \cdot a_{Kout} \qquad \text{(S5b)}$$

The rate constants with index "1" are the rate constants for a_K = 1 mM. The rather large single-channel conductivity of Kcv_{NTS} (ca. 140 pS for I_{true} at symmetrical 500 mM KCl in Fig. 2c in the linear range between -60 mV and +60 mV) argues for a large cytosolic opening with only little diffusion limitation[3]. Thus, it is likely that a_K in the channel's cavity is equal to the cytosolic K^+ activity. However, possible effects of diffusion from the cytosol to the cavity are of minor concern here, since they are included in the value of $k_{12,1}$. Corresponding considerations hold for k_{54}.

Here, we make only those transitions voltage-sensitive where two ions are moved simultaneously through the selectivity filter. The effect of voltage on the binding reactions is expected to be small, since 80% of the voltage is assumed to drop over the selectivity filter[4,5,6].

$$k_{23} = k_{23,0} \exp(s_{23} V / V_{23}) \qquad \text{(S6a)}$$

$$k_{32} = k_{32,0} \exp((s_{23} - 1) V / V_{23}) \qquad \text{(S6b)}$$

and

$$k_{51} = k_{51,0} \exp(s_{51} V / V_{51}) \qquad \text{(S7a)}$$

$$k_{15} = k_{15,0} \exp((s_{51} - 1) V / V_{51}) \qquad \text{(S7b)}$$

s_{ij} are the locations of the Eyring barriers and V_{ij} the characteristic voltages (causing an e-fold increase) of the respective state transitions. The rate constants with index "0" are the rate constants at 0 mV.

Supplementary Table 1. The conversion of K^+ concentrations to activities a_K.

Supplementary Table S1. Conversion of K^+ concentrations to activities a_K[7].

$[K^+]$/mM	20	50	75	100	250	500	750	1000	1500
a_K/mM	17	41	59	77	175	324	465	602	872

Supplementary Note 1: Choice of the current for fitting the IV curves, I_{OF} or I_{true}

The probability for a transition from the open state O to the closed state M as given by k_{OM} depends on the ion occupation prior to this transition. Before a k_{OM} transition, the channel is not open all the time, but subject to O-F gating with much shorter dwell times than the inverse rate constant k_{OM} of 10 ms to 40 µs. Thus, we can assume that the protein averages over the O-F gating with dwell times in the closed state between 1 and 5 µs and a low occupation probability of 10-20%. Therefore, it seems plausible that the average current I_{OF} (Supplementary Eq. S1) determines the state of the protein modulating the O-M transition.

However, this conclusion needs some comments. First, a constant factor of all rate constants as introduced by a constant scaling factor of current in Eq. 1 or Supplementary Eq. S3 is canceled out in Eq. 4 for the P_m. Figure 2c shows that I_{OF} and I_{true} are nearly identical, and the difference results from a constant factor as caused by the negligible voltage dependence of k_{OF} and k_{FO} (Supplementary Eq. S1).

Second, the O-F gating implies that the P_m are different in the closed and open state. Thus, the protein averages over the effects of different P_m related to F and O. Since there is no current in the F-state, IV curve analysis cannot be used to determine the P_m in the state F. What can be done is a rough estimation. The gate for the O-F gating is very likely on the cytosolic side of S0. In the case of BK channels[8] and Kcv_{PBCV-1}[9], it was assumed that the S2 binding site was involved as indicated by MD-simulations[10–12] and crystal structure analysis[13]. An F-gate at the internal side of S0 implies that during its closure negative voltage cannot suck the ions away from S0. Thus, in the closed state F the ion concentration at S0 cannot change too much with voltage and is likely to be close to that at 0 mV.

Even if the above consideration is not completely correct, it has to be taken into account that the dwell time in state F is just 10 to 20% of the dwell time in the open state. Thus, any small errors in the above estimations locating the effective current somewhere between I_{OF} and I_{true} would be negligible in the light of the fact that k_{OM} changes by more than two orders of magnitude. Because of the above considerations, I_{OF} is adequate for the determination of the effective ion occupation from the IV curves, and it is also determined with higher accuracy than I_{true}[14].

Supplementary Note 2: Reliability of the global model-based fits

The global fits start from two different sets of the free model parameters in Fig. 5a. They are taken from the results of preliminary global fits in Origin® of the IV curves alone (Supplementary Eqs. 1 and S3) and of the P_m or $1/P_m$ curves alone (Eq. 5a,b, m = 1, 2, 3, 4, 5). The final global fit of all IV curves and k_{OM} curves together has been done in MATLAB resulting in the numerical values in Supplementary Table S2.

The reliability of the fits has been tested by attributing different relative weights to the IV curves and the ln(k_{OM}) curves with $k_{OM} = w \; P_4/P_3$ (Eq. 6). Table S2 shows the values of the rate constants of ion hopping in the 5-state model in Fig. 5a[1] as obtained from averaging the results from 4 global fits with the relative weights of 0.5, 1, 3 and 10 of the k_{OM} curves. If the ln(k_{OM}) curves have a weight 0.3 as compared to the weighting factor 1 of the IV curves, the fit fails, noticeable by a complete lack of curve match for k_{OM} (not shown). The last column in Table S2 shows the value obtained for a weighting factor of 1.

The scatter factor (geometric mean) in Table S2 shows the error in the rate constants resulting from these different weights. Whereas nearly all parameters are very reliable, $k_{12,1}$ and k_{21} are not well

determined. In tests, k_{12} and k_{21} were found to need a minimum value of 100 (µs*mM)$^{-1}$ and 60000 (µs)$^{-1}$, respectively, but much higher values also gave a good fit (not shown). Thus, their scatter factors are omitted in Table S2. However, for the calculation of the ion occupancies P_m (Eq. 4), only the ratio $k_{12,1}/k_{21}$ is important, and this has an error of only 14% (last row in Supplementary Table S2).

Supplementary Table S2. Results of the global fit of the voltage and K^+ dependence of current and of the rate constant of O-M channel closure (k_{OM}) with the model in Fig. 5a. The geometric mean values are determined from four global fits with different weighting factors (0.5, 1, 3 and 10) adjusting the weight of the ln(k_{OM}) curves relative to the IV curves. The error is given as geometrical error (scatter factor, which gives the standard deviation as a factor) to ease the comparison between the different parameters. "calc" indicates that k_{21} is not a fit parameter as it can be calculated from micro reversibility at 1 mM K^+ on either side and V = 0 mV: $k_{21} = k_{12,0}\, k_{23,0}\, k_{34}\, k_{45}\, k_{51,0} /(k_{32,0}\, k_{43}\, k_{54,0}\, k_{15,0})$. The rate constants $k_{12,1}$ and k_{21} are labelled by *, because these values are not well determined. Only the ratio $k_{12,1}/k_{21}$ (last row) is reliable. The last column gives the parameters of the global fit of current and ln(k_{OM}) with weighting factor 1. The corresponding curves of this fit are displayed in Fig. 6.

**parameter**	Unit	Occurring in Equation	geometric mean	scatter factor	weighting factor 1 for Fig. 6
**$k_{12,1}$ (a_{Kin})**	µs^{-1} mM^{-1}	Eq. S5a	105*		127
**k_{21} (*calc*)**	µs^{-1}	See Title	59768*		66600
**$k_{23,0}$ (V)**	µs^{-1}	Eq. S6a	1214	1.08	1281
**$k_{32,0}$ (V)**	µs^{-1}	Eq. S6b	1941	1.38	2418
**k_{34}**	µs^{-1}	Eq. S3	252	1.12	274
**k_{43}**	µs^{-1}	Eq. S3	685	1.21	613
**k_{45}**	µs^{-1}	Eq. S3	6824	2.49	3658
**$k_{54,1}$ (a_{Kout})**	µs^{-1} mM^{-1}	Eq. S5b	35.4	2.07	22
**$k_{51,0}$ (V)**	µs^{-1}	Eq. S7a	1694	1.04	1689
**$k_{15,0}$ (V)**	µs^{-1}	Eq. S7b	132.6	1.05	129
**s_{23}**		Eq. S6	0.24	1.30	0.27
**V_{23}**	mV	Eq. S6	55.9	1.04	57
**s_{15}**		Eq. S7	0.40	1.11	0.37
**V_{15}**	mV	Eq. S7	44.9	1.06	43
**W**	µs^{-1}	Eq. 7	952	1.38	769
**$k_{45}/k_{54,1}$**			193	1.21	170
**$k_{12,1}/k_{21}$**			0.0018	1.14	0.0019

The fits of the IV curves in Fig. 6 are very good. In the case of k_{OM}, the deviation of two data points at -160 mV with 50 mM internal KCl (green in Fig. 6e) is likely caused by the strong gating merging the closed peak and the open peak (similar to Fig. 3d), thus making the characteristics of the amplitude histogram less obvious to the fitting routine. A similar deviation is also found for varying the external concentration (Fig. 6f). However, the basic feature that the different curves merge at high negative voltages is clearly indicated by the theoretical and the measured curves, as discussed below. Nevertheless, fitting the k_{OM} curves with ion occupation probabilities other than P_4/P_3 leads to worse fits as indicated by the error sums in Table 1 and by some typical examples (Supplementary Figs. S1 and S2).

Supplementary Note 3: Typical examples for the failure of fits with other patterns of ion occupancies than P_4/P_3 in the 5-state model of Fig. 5a

Here, we show some typical global fits based on the 5-state model in Fig. 5a, which show that fits not using P_4/P_3 for the k_{OM} curves cannot fit the data.

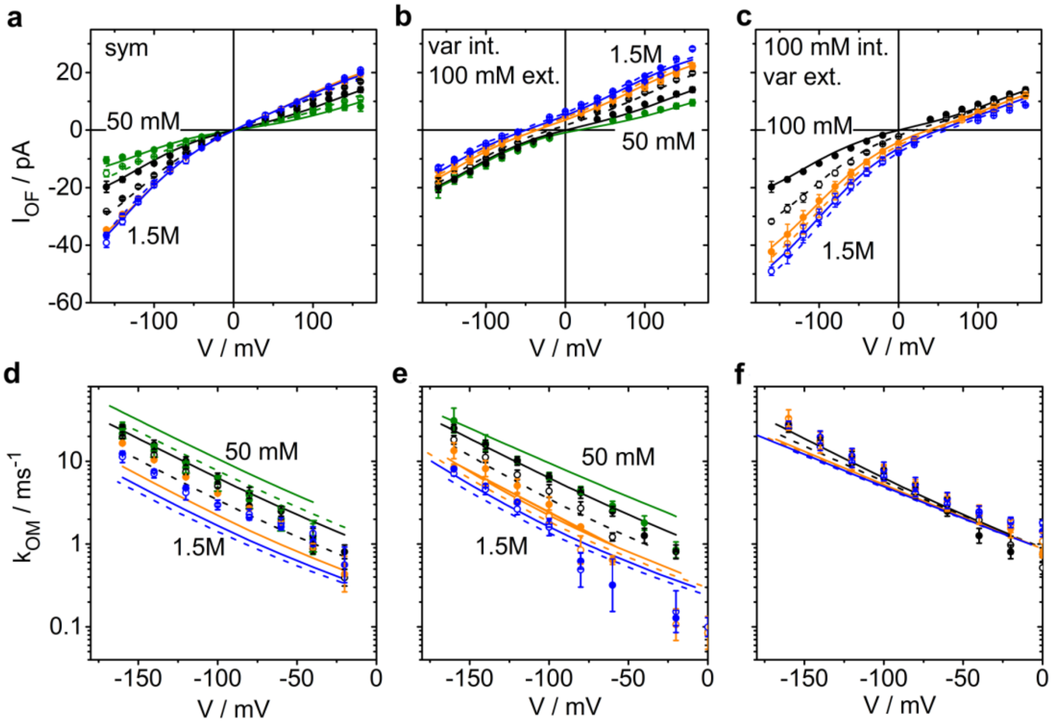

Supplementary Figure S1. The global fit of the IV curves and $k_{OM} = w/P_3$ using the model in Fig. 5a[1]. For color legend and other details, see legend of Figure 6 in the main text.

The global fit of the IV curves with k_{OM} assigned to a single P_m results already in 60 graphs, because in each fit k_{OM} is assigned to a single P_m (the pattern ion concentration) out of those defined by the 10 equations of Eq. 5a,b (m = 1 to 5) with KCl concentration being changed symmetrically or changed only on the external or the internal side. The number of graphs increases to 240 if we consider two partners as in P_4/P_3. Of course, we do not show all of them, but only three representatives for three different values of the error sum in Table 1 (Supplementary Figs. S1 and S2).

The fits shown in Supplementary Figs. S1 and S2 all yield reasonably good fits only of the IV curves. The global fit of current and $k_{OM} = w/P_3$ (Supplementary Fig. S1) is the best fit obtained with a single pattern of ion distribution, namely $1/P_3$. The IV curves are fitted quite well, and the voltage dependence of k_{OM} is also reproduced. However, the dependence on K^+ concentrations is poorly fitted by this hypothesis. This yields the necessity to include P_4 (Eq. 6 and Fig. 6).

If $k_{OM} = w/P_2$ is used, the voltage dependence of k_{OM} is too weak (Supplementary Fig. S2d). The worst case is obtained with $k_{OM} = wP_1$ (Supplementary Fig. S2c). The examples in Supplementary Figs. S1 and S2 visualize the message of the error sums in Table 1, namely, that $k_{OM} = wP_4/P_3$ (Eq. 6) is the only hypothesis, which is consistent with the measured data.

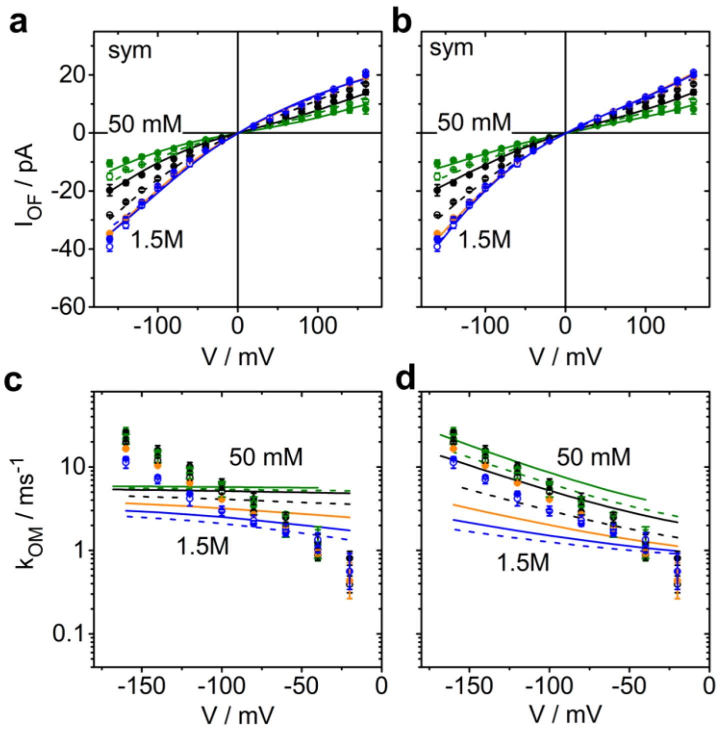

Supplementary Figure S2. Examples for the bad global fits of current and k_{OM} at symmetrical concentrations. **(a,c)** The global fit of the IV curves and $k_{OM} = w*P_1$ represents those with an error sum around 1000. **(b,d)** The global fit of the IV curves and $k_{OM} = w/P_2$ represents those with an error sum around 550 (Table 1). For color legend and other details, see legend of Figure 6 in the main text.

Supplementary Note 4: Numerical peculiarities resulting in the dependence of k_{OM} on *Kmf* (voltage minus Nernst potential) in Fig. 4e

In Fig. 4e of the main text, it is shown that k_{OM} at negative voltages depends on the "K^+ motive force" ($Kmf = V - E_K$) only when internal K^+ concentration is varied. In order to understand how this is predicted by our model (Eq. 6) in quantitative terms, we inspect the dominant terms in the matrices D_4^{C5} and D_3^{C5}. In Supplementary Eq. S8, the values for the rate constants are given at V = -100 mV, a_{Kin} = 602 mM, (i.e. $[K^+_{in}]$ = 1000 mM) and a_{Kout} = 77 mM (i.e. $[K^+_{out}]$ = 100 mM). Supplementary Equation S8 is written as a modified arrow scheme[2] (Eq. S4): Arrows have been replaced by the respective values of the rate constants or with other words: the numbers are the rate constants between the states given in the top line with $k_{left,right}$ above the diagonal and $k_{right,left}$ below the diagonal . The originally empty diagonals of the arrow scheme (Supplementary Eq. S4) are replaced by "1". The columns "product" contain the products of rate constants in the respective rows.

$$\frac{D_4^{C5}}{D_3^{C5}} = \frac{\begin{array}{cccccc} 4 & 5 & 1 & 2 & 3 & 4 \end{array} \left[\begin{array}{ccccc} 1 & 695 & 63210 & 790 & 252 \\ 2726 & 1 & 63210 & 790 & 252 \\ 2726 & 501 & 1 & 790 & 252 \\ 2726 & 501 & 59786 & 1 & 252 \\ 2726 & 501 & 59786 & 7563 & 1 \end{array}\right]}{\begin{array}{cccccc} 3 & 4 & 5 & 1 & 2 & 3 \end{array} \left[\begin{array}{ccccc} 1 & 6824 & 251 & 63210 & 790 \\ 685 & 1 & 251 & 63210 & 790 \\ 685 & 2726 & 1 & 63210 & 790 \\ 685 & 2726 & 501 & 1 & 790 \\ 685 & 2726 & 501 & 59786 & 1 \end{array}\right]} = \frac{\text{product} \begin{bmatrix} 8.7E+12 \\ 3.4E+13 \\ 2.7E+11 \\ 2.0E+13 \\ 6.2E+14 \end{bmatrix}}{\text{product} \begin{bmatrix} 2.4E+14 \\ 2.3E+13 \\ 9.3E+13 \\ 7.6E+11 \\ 5.6E+13 \end{bmatrix}} \quad \text{(S8)}$$

Rate constants printed in black are constant. Voltage dependence is indicated in red (increasing with positive voltage) and in blue (increasing with negative voltage). Green rate constants include the external (77 mM) and magenta rate constants the internal (602 mM) K^+ activity, respectively.

The fifth row is the dominant row in D_4^{C5} as indicated by the values of the products on the right-hand side of the equation. It is of the type *constant*exp [(S$_{51}$-1) V/V$_{51}$ + (s$_{23}$-1) V/V$_{23}$].* a_{Kout} (green) is included in the constant factor because it is kept constant when internal K^+ concentrations is varied. The dominant terms of D_3^{C5} are the rows 1 and 3. Thus, P_3/P_4 can be approximated by

$$\frac{P4}{P3} \approx \frac{k_{54}k_{15}k_{21}k_{32}}{k_{45}k_{51}k_{12}k_{23} + k_{43}k_{54}k_{12}k_{23}} = \frac{k_{54}k_{15,0}k_{21}k_{32,0}\exp((s_{51}-1)V/V_{51})\exp((s_{23}-1)V/V_{23})}{[k_{45}\,k_{51,0}\exp(s_{51}V/V_{51}) + k_{43}k_{54}]\cdot a_{Kin}\,k_{12,1}k_{23,0}\exp(s_{23}V/V_{23})} \quad \text{(S9)}$$

After extraction of the dependence on V and a_{Kin}, all terms in Supplementary Eq. S9 of the type k_{ij} *and* $k_{ij,x}$ (x = 0 or 1) are constant. For the sake of clarity, they are comprised in $const_n$, $const_{d2}$ and $const_{d1}$ as defined by the comparison of Supplementary Eqs. S9 and S10

$$\frac{P4}{P3} \approx \frac{const_n \exp[(s_{51}-1)V/V_{51}]\exp[(s_{23}-1)V/V_{23}]}{const_{d1}\,a_{Kin}\exp[s_{51}V/V_{51}]\exp[s_{23}V/V_{23}] + const_{d2}\,a_{Kin}\exp[s_{23}V/V_{23}]} = \frac{const_n}{const_{d1}\,a_{Kin}\exp[V(1/V_{15}+1/V_{23})] + const_{d2}\,a_{Kin}\exp[V((1-s_{51})/V_{51}+1/V_{23})]} \quad \text{(S10)}$$

Because the dependence on *Kmf* is only observed when a_{Kout} is constant and only a_{Kin} is varied, we take it out of the constant factors to formally introduce the Nernst potential E_K

$$\frac{P4}{P3} \approx \frac{const_n}{const_{d1*}\,a_{Kout}\frac{a_{Kin}}{a_{Kout}}\exp[V(1/V_{51}+1/V_{23})] + const_{d2*}\,a_{Kout}\frac{a_{Kin}}{a_{Kout}}\exp[V((1-s_{51})/V_{51}+1/V_{23})]} \quad \text{(S11)}$$

$$E_K = \frac{RT}{F}\ln\frac{a_{Kout}}{a_{Kin}} \quad \text{(S12)}$$

With the new constant *const* = $const_n/const_{d2}$ and introducing the Nernst potential E_K (Supplementary Eq. S12), Supplementary Eq. S11 is converted to the *Kmf* (K^+-motive force or chemiosmotic potential = *V* - E_K) form

$$\frac{P_4}{P_3} \approx$$

$$\frac{const}{\dfrac{const_{d1}}{const_{d2}} \exp\left[V\left(1/V_{15}+1/V_{23}\right)-E_K\right]+\exp\left[V((1-s_{15})/V_{15}+1/V_{23}-E_K)\right]} \qquad (\text{S13})$$

$$= \frac{const}{\dfrac{const_{d1}}{const_{d2}} \cdot \exp[Kmf_1]+\exp[Kmf_2]}$$

Supplementary Equation S13 shows that in the exponential functions there is a sum of $-E_K$ (Supplementary Eq. S12) and a fraction of the voltage, as determined by *s*. This is the basis for the dependency on the *KmF*, which holds in the case of varying internal [K^+] as found in the inset of Fig. 4e. There are some deviations from a strict dependence on *KmF*, because in Supplementary Eq. S13 two slightly different *KmFs* are involved, and because the smaller terms in Supplementary Eq. S8 are ignored. Nevertheless, the good fit in Fig. 6e shows that the dependence on *Kmf* is a good approximation, and that it originates from the factor P_4/P_3.

Supplementary Note 5: The absence of an effect of external K$^+$ concentration even though the sensor S0 is at the outside

The absence of an effect of external K^+ concentration on k_{OM} (Fig. 4f) at negative voltages comes as a surprise because S0 seems to be the voltage sensor being close to the outside concentration. This can be explained by an inspection of the pattern of ion occupations P_3 and P_4 in Fig. 7 suggesting a mutual compensation of the sensitivity of P_3 and P_4 to external K^+. Here, it is explained in quantitative terms. In Supplementary Eq. S14, the values for the rate constants are given at V = -100 mV, a_{Kin} = 77 mM i.e. [K_{in}] = 100 mM and a_{Kout} = 602 mM i.e. [K_{out}] = 1000 mM.

$$\frac{D_4^{C5}}{D_3^{C5}} = \frac{\begin{array}{ccccc} \multicolumn{5}{c}{4 \quad 5 \quad 1 \quad 2 \quad 3 \quad 4} \\ \left[\begin{array}{rrrrr} 1 & 695 & 8085 & 790 & 252 \\ 21311 & 1 & 8085 & 790 & 252 \\ 21311 & 501 & 1 & 790 & 252 \\ 21311 & 501 & 59786 & 1 & 252 \\ 21311 & 501 & 59786 & 7563 & 1 \end{array}\right] \end{array}}{\begin{array}{ccccc} \multicolumn{5}{c}{3 \quad 4 \quad 5 \quad 1 \quad 2 \quad 3} \\ \left[\begin{array}{rrrrr} 1 & 6824 & 251 & 8085 & 790 \\ 685 & 1 & 251 & 8085 & 790 \\ 685 & 21311 & 1 & 8085 & 790 \\ 685 & 21311 & 501 & 1 & 790 \\ 685 & 21311 & 501 & 59786 & 1 \end{array}\right] \end{array}} = \frac{\begin{array}{c} \text{product} \\ \left[\begin{array}{c} 1.2E+12 \\ 3.4E+13 \\ 2.2E+12 \\ 1.6E+14 \\ 4.9E+15 \end{array}\right] \end{array}}{\begin{array}{c} \text{product} \\ \left[\begin{array}{c} 3.0E+13 \\ 3.0E+12 \\ 9.3E+13 \\ 5.8E+12 \\ 4.4E+14 \end{array}\right] \end{array}} \qquad (\text{S14})$$

The scheme in Supplementary Eq. S14 shows that in the dominant terms the rate constant $k_{45} = k_{45,1}\, a_{Kout}$ (green) occurs as a common factor in the numerator and in the denominator. Thus, a_{Kout} is canceled out in the dominant terms. This explains the much smaller influence of a_{Kout} on k_{OM} as compared to a_{Kin} as shown in Figs. 4f and 6f.

Supplementary Note 6: Global fits with the 4-state model of ion-hopping in Fig. 5b

Reducing the 5-state model in Fig. 5a[1] to the 4-state model in Fig. 5b leads to the omission of state 4 in Fig. 5a. The equations are nearly the same as for Fig. 5a with the exception that the rate constants between state 3 and 4 are omitted. Thus, k_{34} and k_{43} in the 4-state model take over the function of k_{45} and k_{54} of the 5-state model.

Similar to the 5-state model, the global fit of the IV curves and of $\ln(w/P_3)$ is the only fit providing the voltage dependence of the rate constant k_{OM} (Supplementary Fig. S3). However, there is no candidate such as P_4 in the 5-state model, which can account for the dependence on K^+ concentration.

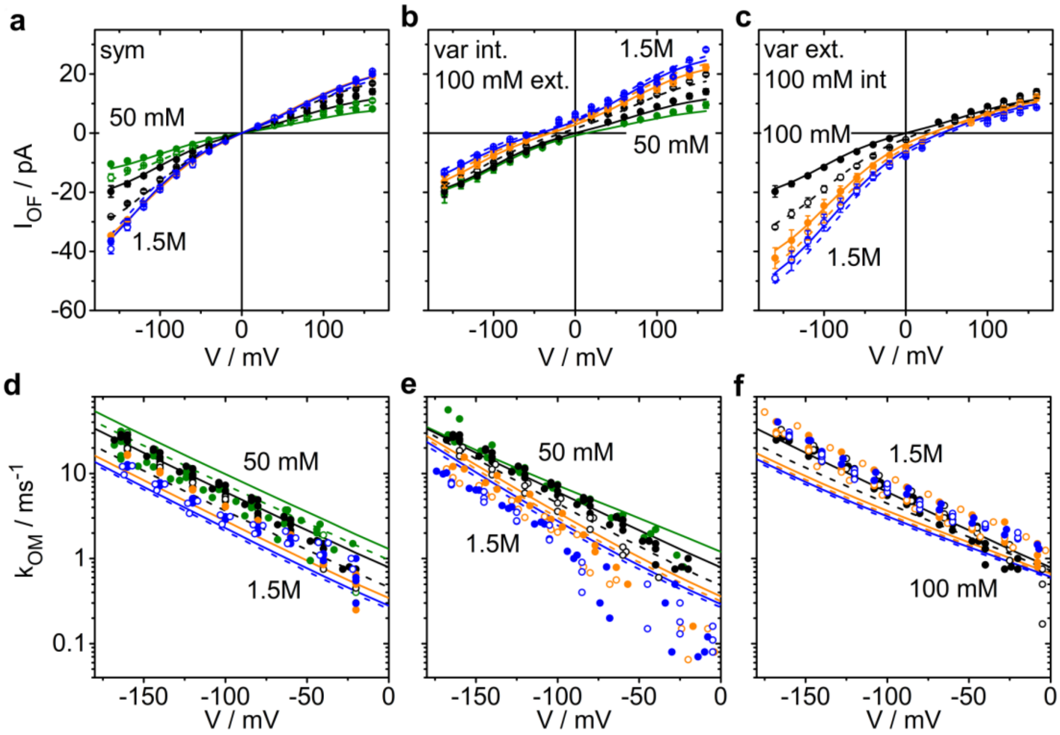

Supplementary Figure S3. The best global fit of the IV curves and k_{OM} (with $\ln(w/P_3)$) in Fig.4 on the basis of the 4-state model in Fig. 5b. For color legend and other details, see legend of Figure 6 in the main text.

Supplementary Note 7: Global fits with the hard knock-on model

The equation for the current in the hard knock-on model of Fig. 5c is the same as Supplementary Eq. S3, but the equations for the individual rate constants are adapted to this model with:

$$k_{12} = k_{12,1} \cdot a_{Kin} \quad \text{(S15a)}$$

$$k_{43} = k_{43,1} \cdot a_{Kout} \quad \text{(S15b)}$$

$$k_{45} = k_{45,0} \exp(s_{45} V / V_{45}) \quad \text{(S16a)}$$

$$k_{54} = k_{54,0} \exp((s_{45} - 1) V / V_{45}) \quad \text{(S16b)}$$

$$k_{51} = k_{51,0} \exp(s_{51} V / V_{51}) \quad \text{(S17a)}$$

$$k_{15} = k_{15,0} \exp((s_{51} - 1) V / V_{51}) \quad \text{(S17b)}$$

Supplementary Figs. S4 and S5 show global fits of the IV curves and rate constant $k_{OM}(V)$ (Fig. 4) on the basis of the model in Fig. 5c. The general problem is that the fits of the IV curves are quite good (Supplementary Fig. S4a to c), but the steepness of the voltage-dependence of k_{OM} is by far not reached (Supplementary Fig. S5d to f). Supplementary Fig. S4a to f shows the "best" fit result in a global fit of the IV curves and ln(1/P_5).

At least the rough trend of this K^+ dependence (direction and difference between internal and external concentration) could be modelled in a global fit of the IV curves and ln(1/P_2). Again, the fit of the IV curves is not distinguishable from that in Fig. 6a to c, but the steepness of the voltage-dependency of k_{OM} (Supplementary Fig. S5d to f) is even worse than that in Supplementary Fig. S4d to f.

However, the message of these fits is that the hard-knock on model completely fails to explain the voltage and K^+ dependency of k_{OM} by means of voltage-dependent ion occupation in the selectivity filter.

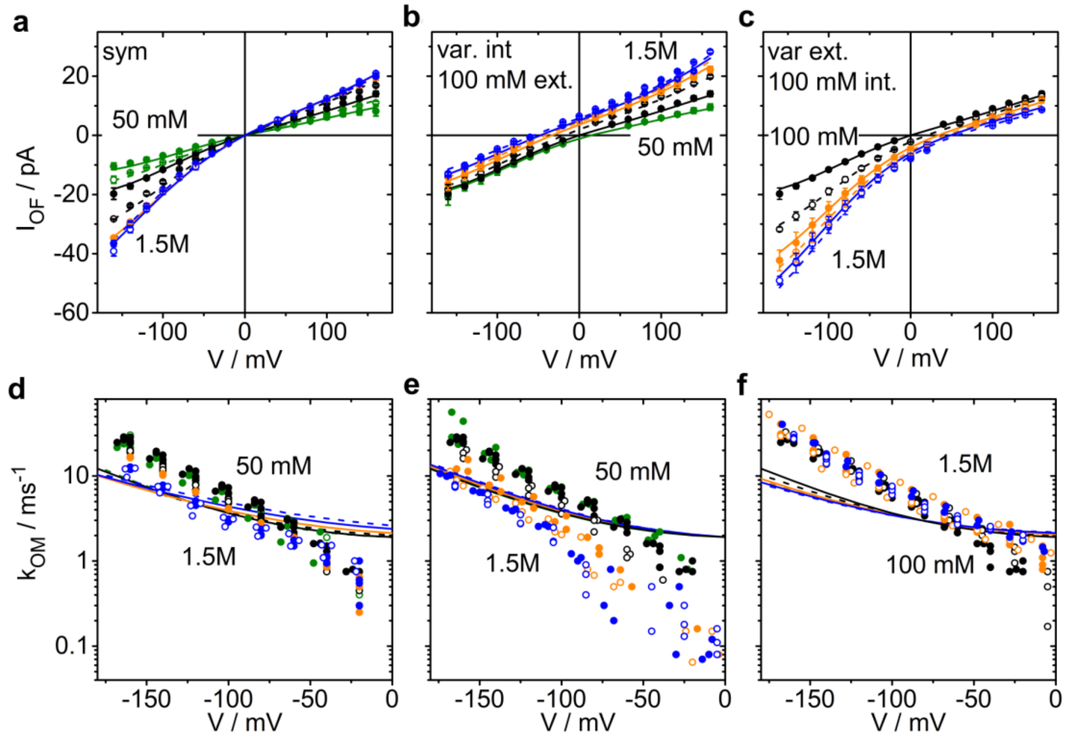

Supplementary Figure S4. Best fit for a single occupation state for the hard-knock on model (Fig. 5c). k_{OM} was fitted with w/P_5. For color legend and other details, see legend of Figure 6 in the main text.

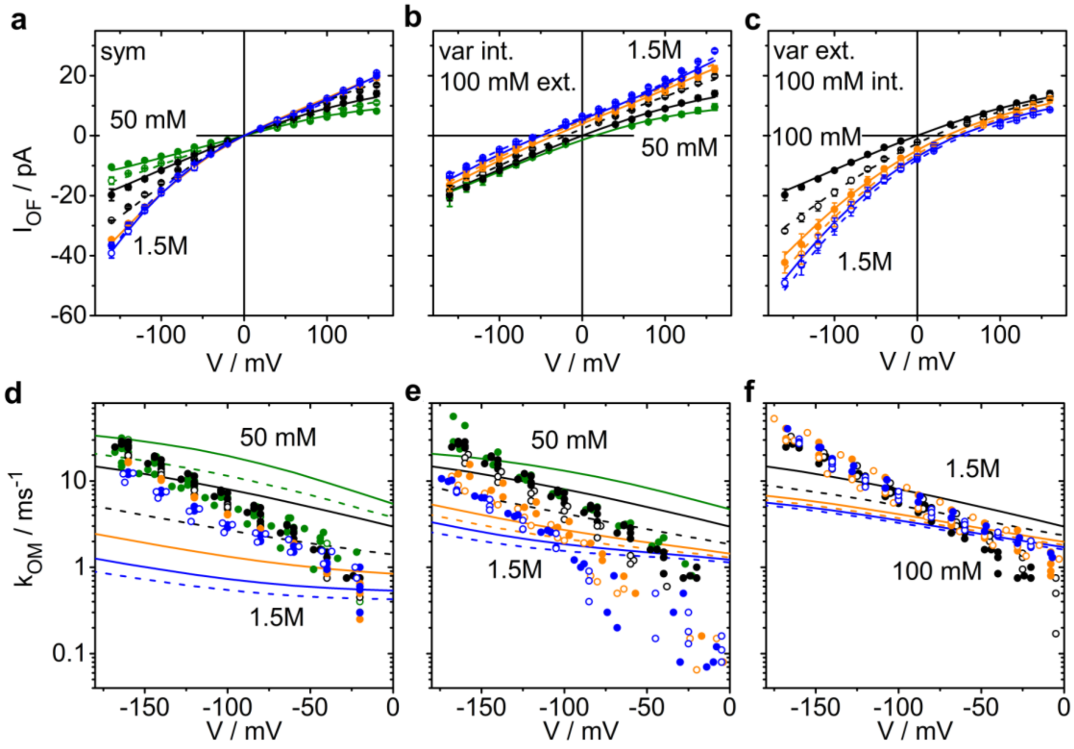

Supplementary Figure S5. Another fit for a single occupation state for the hard-knock on model (Fig. 5c). k_{OM} was fitted with w/P_2. Not more than a rough trend of the K^+ dependence could be achieved. For color legend and other details, see legend of Figure 6 in the main text.

Supplementary Note 8: The lab-made program bownhill.exe

The program bownhill.exe, the auxiliary files and a help-file can be downloaded from http://www.bio.tu-darmstadt.de/ag/professuren/indraschroeder/software.en.jsp together with the data for a demonstrative run.

Supplementary Figure S6: The flow diagram of the global fit

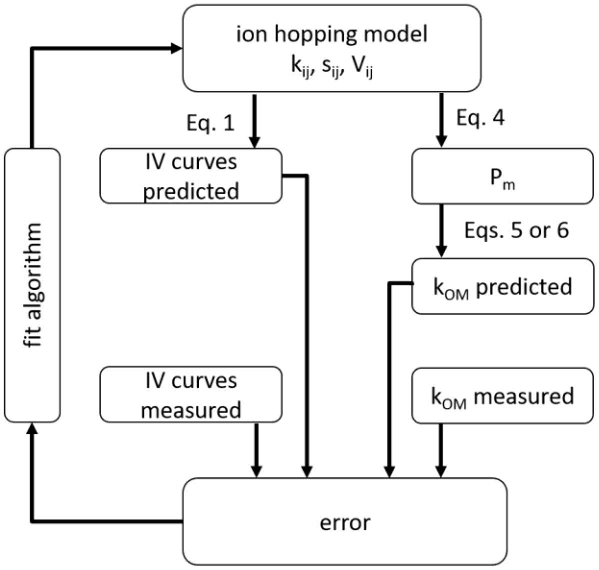

Supporting References

1. Roux, B. Ion conduction and selectivity in K^+ channels. *Annu. Rev. Biophys. Biomol. Struct.* **34,** 153–171 (2005).
2. Hansen, U.-P., Rauh, O. & Schroeder, I. A simple recipe for setting up the flux equations of cyclic and linear reaction schemes of ion transport with a high number of states: the arrow scheme. *Channels* **10,** 1–20 (2016).
3. Naranjo, D., Moldenhauer, H., Pincuntureo, M. & Díaz-Franulic, I. Pore size matters for potassium channel conductance. *J. Gen. Physiol.* **148,** 277–291 (2016).
4. Jiang, Y. *et al.* The open pore conformation of potassium channels. *Nature* **417,** 523–546 (2002).
5. Contreras, J. E. *et al.* Voltage profile along the permeation pathway of an open channel. *Biophys. J.* **99,** 2863–2869 (2010).
6. Andersson, A. E. V., Kasimova, M. A. & Delemotte, L. Exploring the viral channel $Kcv_{PBCV\text{-}1}$ function via computation. *J. Membr. Biol.* **in press,** (2018).
7. Hamer, W. J. & Wu, Y.-C. Osmotic coefficients and mean activity coefficients of uni-univalent electrolytes in water at 25°C. *J. Phys. Chem. Ref. Data* **1,** 1047–1100 (1972).
8. Schroeder, I. & Hansen, U.-P. Saturation and microsecond gating of current indicate depletion-induced instability of the MaxiK selectivity filter. *J. Gen. Physiol.* **130,** 83–97 (2007).
9. Abenavoli, A. *et al.* Fast and slow gating are inherent properties of the pore module of the K^+ channel Kcv. *J. Gen. Physiol.* **134,** 219–229 (2009).
10. Bernèche, S. & Roux, B. A gate in the selectivity filter of potassium channels. *Structure* **13,** 591–600 (2005).
11. Capener, C. E., Proks, P., Ashcroft, F. M. & Sansom, M. S. P. Filter flexibility in a mammalian K channel: models and simulations of Kir6.2 mutants. *Biophys. J.* **84,** 2345–2356 (2003).
12. Miloshevsky, G. V & Jordan, P. C. Conformational changes in the selectivity filter of the open-state KcsA channel: an energy minimization study. *Biophys. J.* **95,** 3239–3251 (2008).
13. Zhou, Y., Morais-Cabral, J. H., Kaufman, A. & MacKinnon, R. Chemistry of ion coordination and hydration revealed by a K^+ channel-Fab complex at 2.0 A resolution. *Nature* **414,** 43–48 (2001).
14. Rauh, O. *et al.* Extended beta distributions open the access to fast gating in bilayer experiments-assigning the voltage-dependent gating to the selectivity filter. *FEBS Lett.* **591,** 3850–3860 (2017).
